# Thermal-Gated Self-Repairing Polyimide Separator for Dendrite-Suppressed Lithium Metal Batteries

**DOI:** 10.1007/s40820-025-02050-2

**Published:** 2026-01-30

**Authors:** Pengpeng Li, Xinluo Li, Yisong Zhou, Yingying Zhang, Nianyu Yue, Jiameng Li, Yumeng Xin, Lianlong Hou, Jiaji Yue, Xin Zhang, Guohua Sun, Nanjun Chen

**Affiliations:** 1 Hebei Key Laboratory of Flexible Functional Materials, College of Materials Science and Engineering, Hebei University of Science and Technology, Shijiazhuang, 050018 People’s Republic of China; 2 State Key Laboratory of Physical Chemistry of Solid Surfaces, College of Chemistry and Chemical Engineering, Xiamen University, Xiamen, 361005 People’s Republic of China; 3 State Key Laboratory of Organic-Inorganic Composites, Beijing University of Chemical Technology, Beijing, 100029 People’s Republic of China

**Keywords:** Lithium metal batteries, Separator, Thermal shutdown, Self-repairing, Dendrite

## Abstract

The internal heat generation and the growth of lithium dendrites have raised severe safety issues in lithium metal batteries (LMBs), which significantly hinder their widespread adoption. Therefore, it is critical to develop intelligent separators to improve the security and performance of LMBs. Here, we engineer a self-repairing polyetherimide (PEI)-functionalized polyamide-imide (PAI@PEI) nanofiber separator with a thermal-gated function, in which the thermoplastic PEI core has an automatically thermal shutdown function via intelligent closure of apertures under high temperature, while the thermosetting PAI shell can drive the remodeling of PEI to restore its apertures. The PAI@PEI separator showcases the topmost aperture-closing temperature of 400 °C compared to the cutting-edge separators that typically have an aperture-closing temperature below 200 °C. Morphological characterization confirms that the PAI@PEI separator with a closed aperture can recover its apertures at 350 °C, endowing the PAI@PEI separator with a unique self-repairing function to enhance the longevity and safety of LMBs. Meanwhile, density functional theory calculations reveal that the polar amide and imide groups in PAI@PEI separator, both before and after aperture restoration, can efficiently facilitate Li-ion dissociation and transportation for suppressing lithium dendrite growth. As a result, the aperture-restored PAI@PEI separator (R-PAI@PEI) demonstrates significantly improved overall electrochemical performance. Specifically, the R-PAI@PEI-based Li||Li cell exhibits an exceptional Li-ion transference number of 0.71 and an excellent cycling stability at 1 mA cm^−2^ for over 750 h, which significantly outperform commercial and state-of-the-art separator-based LMBs (typically below 0.65 and 500 h, respectively). Importantly, the R-PAI@PEI-based Li||NCM523 battery still exhibits an impressive specific capacity of 99.7 mAh g^−1^ at 5C and maintains 90% of its capacity after 100 cycles. These results underscore the feasibility of designing functional separator, opening a new avenue for next-generation highly safe LMBs separators.

**Supplementary Information:**

The online version contains supplementary material available at 10.1007/s40820-025-02050-2.

## Highlights

- The self-repairing polyetherimide (PEI)-functionalized polyamide-imide (PAI@PEI) nanofiber membrane with a thermal-gated function was designed to enhance the thermal safety properties of lithium metal batteries.
- The PAI@PEI membrane with the polar amide and imide groups facilitates Li^+^ dissociation and transport, which is essential for suppressing dendrite growth.
- The aperture-restored PAI@PEI-based Li||Li cell exhibits an exceptional Li^+^ transference number of 0.71 and an excellent cycling stability at 1 mA cm^−2^ for over 750 h.

**Supplementary Information:**

The online version contains supplementary material available at 10.1007/s40820-025-02050-2.

## Graphical abstract

**Figure:**
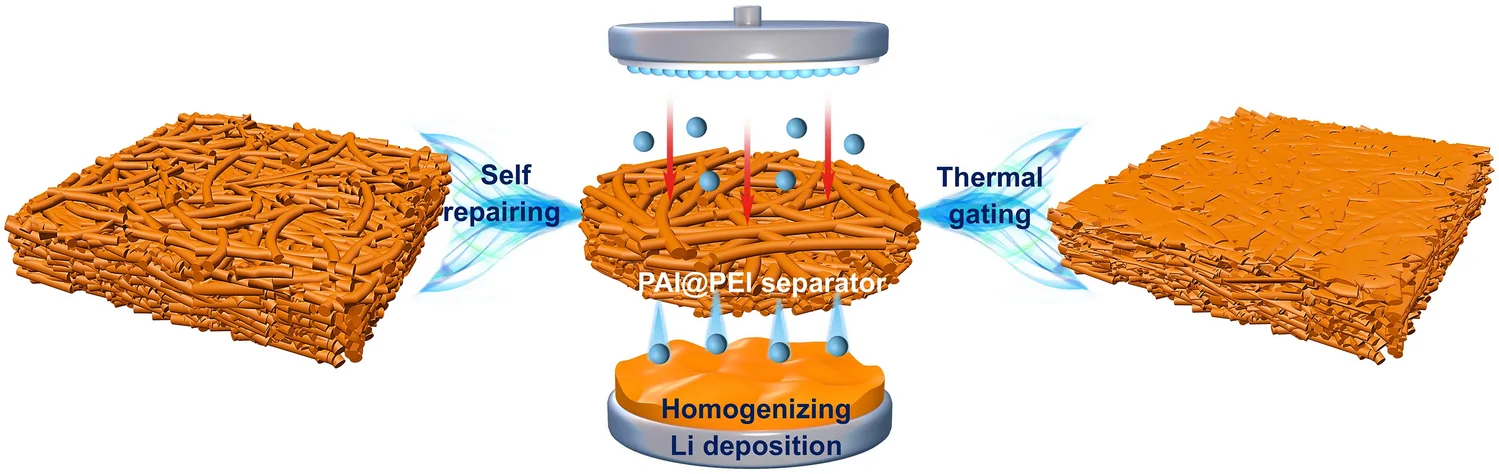

## Introduction

Driven by the global demand for energy efficiency and environmental sustainability, electrochemical energy storage technologies are advancing rapidly with the aim of achieving high capacity, long cycle life, and fast charge rates [1–8]. In this context, lithium metal batteries (LMBs) have gained widespread attention due to their high theoretical specific capacity and low electrochemical potential [9]. However, safety issue remains a critical challenge in practical applications of LMBs [10]. On the one hand, the root cause of safety issues in LMBs is internal heat generation during operation, which can be caused by overcharging, internal short-circuiting, or vehicle collisions, potentially triggering safety accidents [11]. Specifically, separators play a pivotal role in enhancing the safety and performance of LMBs by facilitating efficient ionic transport while preventing direct contact between the anode and cathode [12, 13]. On the other hand, the uncontrolled growth of Li dendrites during LMBs charge/discharge cycles notably impairs the coulombic efficiency (CE) and lifespan of LMBs [14, 15]. The formation of Li dendrites can penetrate polymer-based separator, causing internal short circuit and even thermal runaway or combustion. Currently, polyethylene (PE) and polypropylene (PP) separators are widely used in LMBs due to low cost, electrochemical stability, and acceptable mechanical strength [16]. However, the poor thermal stability of PE and PP poses a critical safety concern to their applications in practical LMBs due to the potential internal short circuit and thermal runaway at elevated temperatures [17]. As a result, the development of heat-resistant functional separators is pivotal to enhance the security and performance of LMBs [18–23].

Considerable efforts have been dedicated to enhancing battery safety by designing the advanced separators with a thermal shutdown capacity, mainly including the polymer melt and organic phase-change materials as an overheating-response layer for closing apertures of separator [24–29]. However, the thermal shutdown function of these separators was irreversible, which means that the apertures of separators were permanently closed, resulting in the inactivation of LMBs [30]. This limitation underscores the necessity for separators with reversible thermal-responsive properties. In addition, Li dendrite penetration remains a pervasive threat, necessitating strategies to homogenize Li deposition [31]. To mitigate this issue, it is essential to regulate Li deposition and prevent uneven growth. Multiple strategies have been developed to mitigate dendrite growth in commercial polyolefin-based separators, including mechanical blocking of Li dendrites, regulation of Li-ion (Li^+^) deposition, and optimization of Li^+^ transport pathways [32–38]. Given the thermal instability and electrolyte incompatibility of polyolefin materials, the development of heat-resistant polymer separators with polar structural units is crucial to suppress Li dendrite formation [39, 40]. However, research on multifunctional separators that integrate both thermal responsiveness and dendrite suppression remains limited.

Here, we engineer a smart polyamideimide shell-encapsulated polyetherimide-core nanofiber separator (PAI@PEI) with a thermal-gated function, wherein the PEI core exhibits an automatic thermal shutdown feature by closing separator’s apertures under high temperature conditions, while the PAI shell facilitates the remodeling of the PEI core to restore its apertures. Therefore, the PAI@PEI separator exhibits an unprecedented aperture-closing temperature of 400 °C, which is significantly higher than that of all conventional separators (typically below 200 °C). Furthermore, scanning electron microscope (SEM) images confirm that the PAI@PEI separator can recover its apertures at temperatures of 350 °C. Simultaneously, the polar amide and imide groups of PAI and PEI can efficiently facilitate Li^+^ dissociation and regulate Li^+^ uniform transport, as evidenced by the strong binding energies with Li^+^ (− 3.29 eV for PAI and − 3.81 eV for PEI) via the density functional theory (DFT) calculations. Attributing to the exceptional merits, the Li||Li cell using a R-PAI@PEI-based separator demonstrates an outstanding Li^+^ transference number of 0.71, along with remarkable cycling stability at 1 mA cm^−2^, lasting for over 750 h. These remarkable properties provide excellent specific capacity (99.7 mAh g^−1^ at 5C) for R-PAI@PEI-based Li||NCM523 batteries, along with 100-cycle battery stability at 1C, maintaining 90% of its capacity.

## Experimental Section

### Preparation of PAI@PEI Nanofiber Membrane

To prepare a homogeneous 12 *wt*% polyamide acid (PAA) solution, 2.1489 g of BPDA was gradually added into a 50 mL DMAc solution containing 1.9570 g of DABA. The mixture was vigorously stirred at 0–10 °C for 48 h, followed by standing for degassing. Meanwhile, a 20 *wt*% polyetherimide (PEI) core solution was prepared by dissolving 5 g of PEI in a 50 mL of *N*-methyl-2-pyrrolidone (NMP) and stirring mechanically at 120 °C for 6 h. Both the PAA shell precursor and PEI core solutions were then allowed to stand before being loaded into separate syringes equipped with stainless-steel needles. Coaxial PAA@PEI nanofiber nonwovens were fabricated via electrospinning. The voltage was adjusted between 25 and 30 kV, and the collector rotation speed was set to 350 rpm. Following electrospinning, the PAA@PEI nanofiber separator was thermally treated at 300 °C for 120 min to yield the final PAI@PEI nanofiber separator.

### Characterization

The morphology of the PAI@PEI fibrous composite films, both before and after hot-pressing, was analyzed using a scanning electron microscope (SEM, Hitachi S-4800-I). The chemical composition of the composites was investigated using Fourier-transform infrared spectroscopy (FT-IR, Nicolet 8700). Mechanical strength and puncture strength were evaluated using a universal tensile testing machine (INSTRON 3344), and the puncture strength test employed a steel needle with a diameter of 1.0 mm and a tip radius of 0.5 mm at a speed of 100 mm min^−1^. The differential scanning calorimetry (DSC, TA Instruments Q200) was employed to assess the melting behavior. Thermal stability was evaluated through thermogravimetric analysis (TGA, TA Instruments Q50) under an air atmosphere. Dimensional stability was measured by thermomechanical analysis (TMA, TA Instruments Q800). Wettability of the electrolyte was quantified using contact angle measurements (OCA20, Data Physics, Germany). Glass transition temperature (*T*_g_) was determined using a DMA (Q800 TA Instruments, USA), with the sample heated from room temperature to 500 °C at 5 °C min^–1^ under nitrogen. The X-ray photoelectron spectroscopy (XPS) was used for evaluating the electrochemical stability of separator during long-term cycling. The air permeability of the separators was measured using a Gurley densometer (GTR-704R II), where the Gurley number, representing the time required for 100 mL of gas to pass through a 6.45 cm^2^ area under 1.21 kPa pressure, was used to evaluate pore connectivity.

### Electrochemical Measurement

The battery tests, based on a Li||separator||Li configuration, were conducted under DC polarization conditions. The exchange current density (*i*_0_) was measured by linearly fitting the Tafel plots at a sweep rate of 1.0 mV s^−1^ from − 200 to 200 mV. The interfacial resistance of the battery was measured using AC impedance. The Li^+^ transference number (*t*_Li_^+^) was determined by the Bruce-Vincent method with an applied potential of *ΔV* = 10 mV for 1800 s, and the result was calculated using Eq. (1):

1 $$t_{{{\mathrm{Li}}^{ + } }} = I_{s} \left( {\left( {\Delta V - I_{0} R_{0} } \right)/I_{0} \left( {\Delta V - I_{s} R_{s} } \right)} \right)$$

where *I*_0_ and *I*_s_ represent the currents at initial and steady states, respectively, while *R*_0_ and *R*_*s*_ denote the interfacial resistances at the initial and steady states. Galvanostatic cycling of Li||Li symmetric cells were conducted at a current density of 1 mA cm^−2^. The polarization voltage of the cells increased progressively with current density, ranging from 0 to 8 mA cm^−2^, with increments of 0.2 mA cm^−2^, to evaluate the critical current density (CCD) of the Li||Li cells. The rate performance of the Li||Li cell was investigated at current densities ranging from 0.5 to 2 mA cm^−2^ with charge/discharge durations of 30 min. Li||Cu cells were assembled to investigate the average coulombic efficiency (CE_avg_), the nucleation overpotential, and the Sand’s time. The charge/discharge performance was systematically assessed at various current rates using a battery testing system (LAND CT2001A). Long-term cycling stability was evaluated at a constant rate of 1C. All coin-type cells (CR2032) were fabricated in an argon-filled glovebox to maintain an inert atmosphere. The commercial cathode material consists of NCM523, carbon black (Super P), graphite (KS-6), and polyvinylidene fluoride (PVDF) as the binder. Additionally, the active material loading was 8.51 mg cm^−2^.

## Results and Discussion

### Design of PAI@PEI Core–shell Nanofibers and Characterization

**Fig. 1:**
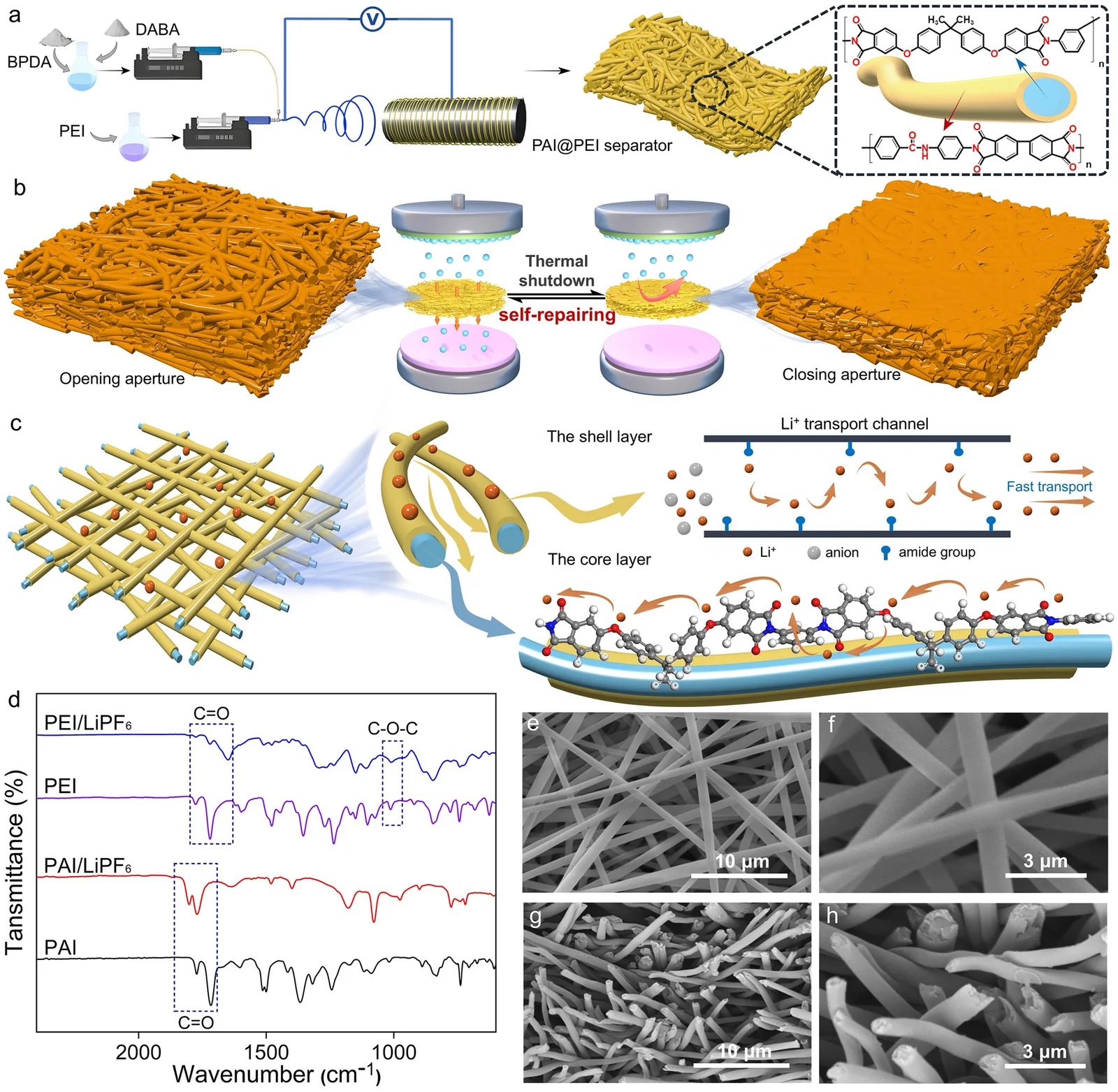
Schematic diagram of synthesis and structural characterization for smart PAI@PEI separator. **a** Preparation of PAI@PEI separator via a coaxial electrospinning strategy. **b** Schematic illustration of thermal response function of the PAI@PEI separator. **c** Schematic diagram of the interaction between Li^+^ with PAI and PEI. **d** FTIR spectra of PAI/LiPF_6_ and PEI/LiPF_6_. **e, f** Surface and **g, h** cross-sectional SEM images of the PAI@PEI membrane

Polyamide-imide (PAI) was synthesized by the polycondensation reaction between dianhydride and diamine (Fig. S1), which is stable below 400 °C due to its thermosetting nature (Fig. S2). Therefore, we devised a core–shell structural PAI@PEI nanofiber separator with a thermal-gated function via electrostatic coaxial co-spinning (Fig. 1a), which employs the commercial PEI as the core and PAI as a shell. This unique design utilizes the thermoplastic PEI core to close apertures for endowing the PAI@PEI nanofiber separator with an automatically thermal shutdown function, while the rebound resilience of PAI facilitates the remodeling of the PEI core to restore separator’s apertures. Specifically, as shown in Fig. 1b, the PAI@PEI membrane shows an intrinsic self-recovery capability via 350 °C-triggered reconfiguration of its apertures, resulting from the shape memory of PAI shell. Significantly, polyimide (PI), a typical example of emerging shape-memory polymer materials, is renowned for its exceptional mechanical properties, thermal stability, and shape memory capabilities [41]. The PAI shell, a derivative of PI, can deform under external pressure at 400 °C and returns to its original shape once the external stress is released at 350 °C, which is close to its glass transition temperature of 371 °C (Fig. S3) [42, 43]. Fourier-transform infrared spectroscopy (FTIR) analysis (Fig. S4) reveals distinct absorption peaks corresponding to the C=O stretch at 1669 cm^−1^ and N–H bond at 3364 cm^−1^ of the PAI amide group. Meanwhile, the PAI@PEI also displays the characteristic peaks of C–O–C (1104 and 1270 cm^−1^) of PEI. These characteristic peaks confirm the successful integration of PEI and PAI within the PAI@PEI separator. Besides, the polar amide and imide groups of PAI and PEI can serve as the adsorption sites of Li^+^ to build transport channel, regulating Li^+^ uniform transport (Fig. 1c). To further examine the interaction between PAI and PEI with LiPF_6_, both materials were immersed into electrolyte solutions (PAI/LiPF_6_ and PEI/LiPF_6_), followed by FTIR analysis (Fig. 1d). The observed attenuation and shifts in the characteristic peaks, particularly *ν*_*s*_ (C=O) and *ν*_*s*_ (C–O–C), signify strong interactions between PAI and PEI with Li^+^ [44]. Consequently, the smart PAI@PEI separator provides abundant adsorption sites for dissociating Li^+^ from the electrolyte, thereby homogenizing Li^+^ flux and further suppressing Li dendrite growth. Besides, the as-prepared PAI@PEI membrane exhibits a randomly arranged and uniformly distributed surface microstructure (Fig. 1e, f), while the cross-sectional SEM images reveal a clear core–shell structure (Fig. 1g, h). Notably, the synergetic effect of PAI chain with a hydrogen bond and the ether bond-containing PEI backbone among the homogeneous PI-based core–shell nanofiber mitigates interface defect. As a result, the PAI@PEI membrane achieves enhanced tensile strength (30 MPa) and elastic modulus (0.70 GPa) (Fig. S5). The high elastic modulus of the PAI@PEI membrane helps to impede the growth of Li dendrites, thus ensuring the secure cycling of LMBs.

### Thermal Response Function and Thermostability

**Fig. 2:**
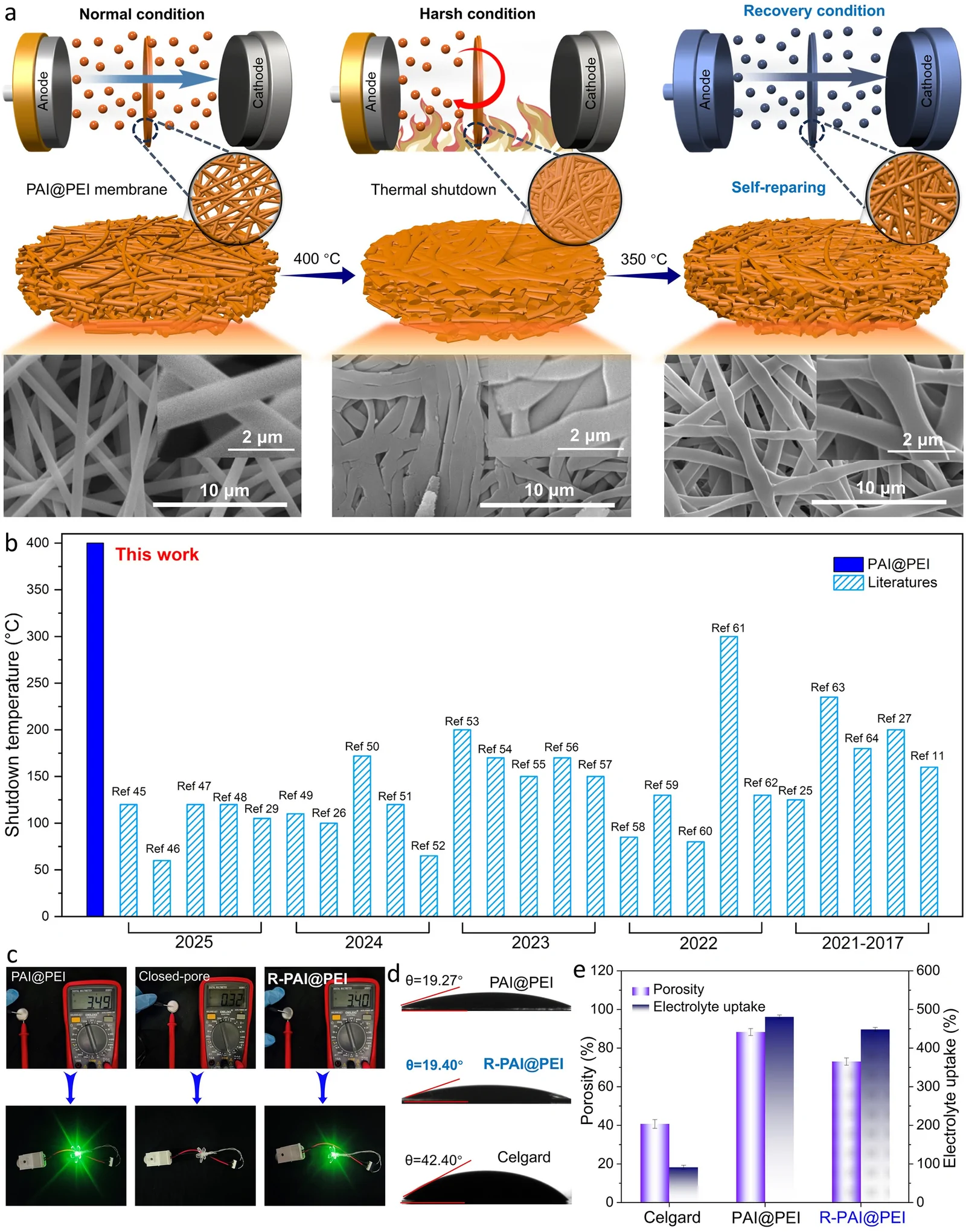
Schematic illustration of thermal response and comparison of shutdown temperature and physical properties. **a** Illustrations of the normal operation, harsh condition, and recovery condition of PAI@PEI separator for LMBs applications.** b** Comparison of shutdown temperature with the state-of-the-art separators [11, 25–27, 29, 45–64]. **c** Digital images of the DC voltage for the PAI@PEI-based Li||NCM523 batteries before and after heat treatment and corresponding LED lighting. **d** Contact angle, **e** electrolyte uptake, and porosity of the R-PAI@PEI, PAI@PEI, and Celgard samples

The thermal-gated function of the PAI@PEI membrane under heat stimulation and specific pressure is illustrated in Fig. 2a. The surface morphology of the PAI@PEI membrane is displayed between 350 and 400 °C, illustrating the gradual transition from open pores to closed apertures (Fig. S6). At 400 °C, the PAI shell extrudes the PEI melt, forming a flat core–shell nanofiber and thus creating a closed-aperture structure. When the closed-aperture PAI@PEI membrane is exposed to temperatures ranging from 320 to 360 °C (Fig. S7), its porous structure gradually undergoes a transformation, leading to the opening of the apertures through a self-driven reconfiguration process. Obviously, the PAI@PEI membrane fully opens its apertures and maintains a compact structure at 350 °C. The PAI@PEI membrane after restoring aperture is simply referred as R-PAI@PEI. This change is attributed to the thermoplastic properties of PEI and the resilience of PAI, and the PAI shell can facilitate the nanofiber’s rebound ability at elevated temperatures, as validated by SEM analysis (Fig. 2a). To further verify the recoverability of the PAI shell, the entire shape memory process of a star-shaped film (in its original shape), made from PAI@PEI, was recorded at high temperature (Fig. S8), clearly showing the recovery process. The schematic diagram of the self-recovering mechanism for PAI@PEI is displayed in Fig. S9. Moreover, the fiber diameters of PAI@PEI, closed-aperture PAI@PEI (C-PAI@PEI), and R-PAI@PEI indirectly confirm the reconfiguration process of the fibers (Fig. S10a–c), and the aperture diameters of the R-PAI@PEI membrane are smaller than those of the PAI@PEI membrane (Fig. S10d, e). Moreover, the opening and closing aperture test reveals the fine self-repairing function of PAI@PEI membrane after heating and cooling cycles (Fig. S11), showing the feasibility of its thermal-gated self-repair behavior in LMBs. After multi-cycle tests, the tensile strength, aperture diameters, puncture strength, porosity, gas permeability, and ionic conductivity of PAI@PEI-based membrane have no significant change (Figs. S5, S12, and Table S1), further verifying the reversible thermal shutdown function. Notably, the PAI@PEI separator, with its reversible thermal shutdown capability, significantly achieves a record closed-aperture temperature of 400 °C, exceeding values reported for cutting-edge separators (Fig. 2b and Table S2, respectively) [11, 25–27, 29, 45–64]. This elevated shut-off temperature not only enhances battery safety at higher temperatures, but also delays the critical point for triggering thermal runaway under extreme conditions such as overcharging, internal short circuits, or external thermal shock, providing a longer response window for safety systems. The DC voltage of the PAI@PEI separator before and after thermal treatment was measured, and both the PAI@PEI and R-PAI@PEI separators can operate normally, as shown in Fig. 2c. Furthermore, the presence of polar amide and imide groups in PAI and PEI endows the PAI@PEI and R-PAI@PEI separators with excellent wettability, as indicated by a low contact angle of 19.27° and 19.40°, as well as rapid electrolyte absorption within 10 s (Figs. 2d and S13, respectively). In contrast, the Celgard membrane exhibits a higher contact angle of 42.4°, with electrolyte droplets maintaining their original shape due to its intrinsic hydrophobicity. Consequently, the PAI@PEI-based separator shows superior electrolyte absorption (Fig. S14). The PAI@PEI and R-PAI@PEI separators also demonstrate improved electrolyte uptake (480.8% and 448.2%, respectively) and porosity (88.3% and 73.0%, respectively) compared to the Celgard membrane, which has electrolyte uptake of 91.1% and porosity of 40.7%, as shown in Fig. 2e. The thermostability of separators is a critical factor in determining the safety of batteries, especially during thermal runaway events, where separator shrinkage or ignition can compromise the battery’s security. Considering the structural consistency of PAI@PEI and R-PAI@PEI, the thermal properties of PAI@PEI were investigated solely for comparison with the performance of the Celgard membrane. Thermogravimetric analysis (TGA) of the PAI@PEI coaxial nanofiber membrane only exhibits a 5% weight loss at 530 °C. In comparison, the Celgard membrane begins to decompose at approximately 240 °C, with complete mass loss occurring by 400 °C (Fig. S15). These results highlight the superior thermal stability of PAI@PEI separator. Further supporting these findings, thermomechanical analysis (TMA) emphasizes the exceptional dimensional stability of the PAI@PEI separator, which maintains its structural integrity up to 300 °C (Fig. S16). In contrast, the Celgard separator experiences significant thermal deformation at around 150 °C due to the softening of the polyolefin matrix. Differential scanning calorimetry (DSC) analysis shows a small melting peak at 220 °C for PAI@PEI separator due to the presence of thermoplastic PEI. This behavior sharply contrasts with the Celgard separator, which shows a distinct melting peak at approximately 168 °C (Fig. S17). The thermal shrinkage of the separators was assessed over a 2 h period at various temperatures. The PAI@PEI separator displays exceptional dimensional stability at 200 °C (Fig. S18). In contrast, the Celgard separator undergoes significant shrinkage and deformation at 150 °C and completely melt at 200 °C. Ignition experiments confirm the superior flame-retardant properties of the PAI@PEI-based separator (Fig. S19), which exhibits self-extinguishing behavior due to its aromatic heterocyclic structure. In contrast, the polyolefin-based Celgard separator ignite rapidly and melt upon flame exposure. These findings underscore the superior thermal stability and flame resistance of the PAI@PEI separator, highlighting its potential for enhancing the safety of LMBs.

### Electrochemical Performances Test

**Fig. 3:**
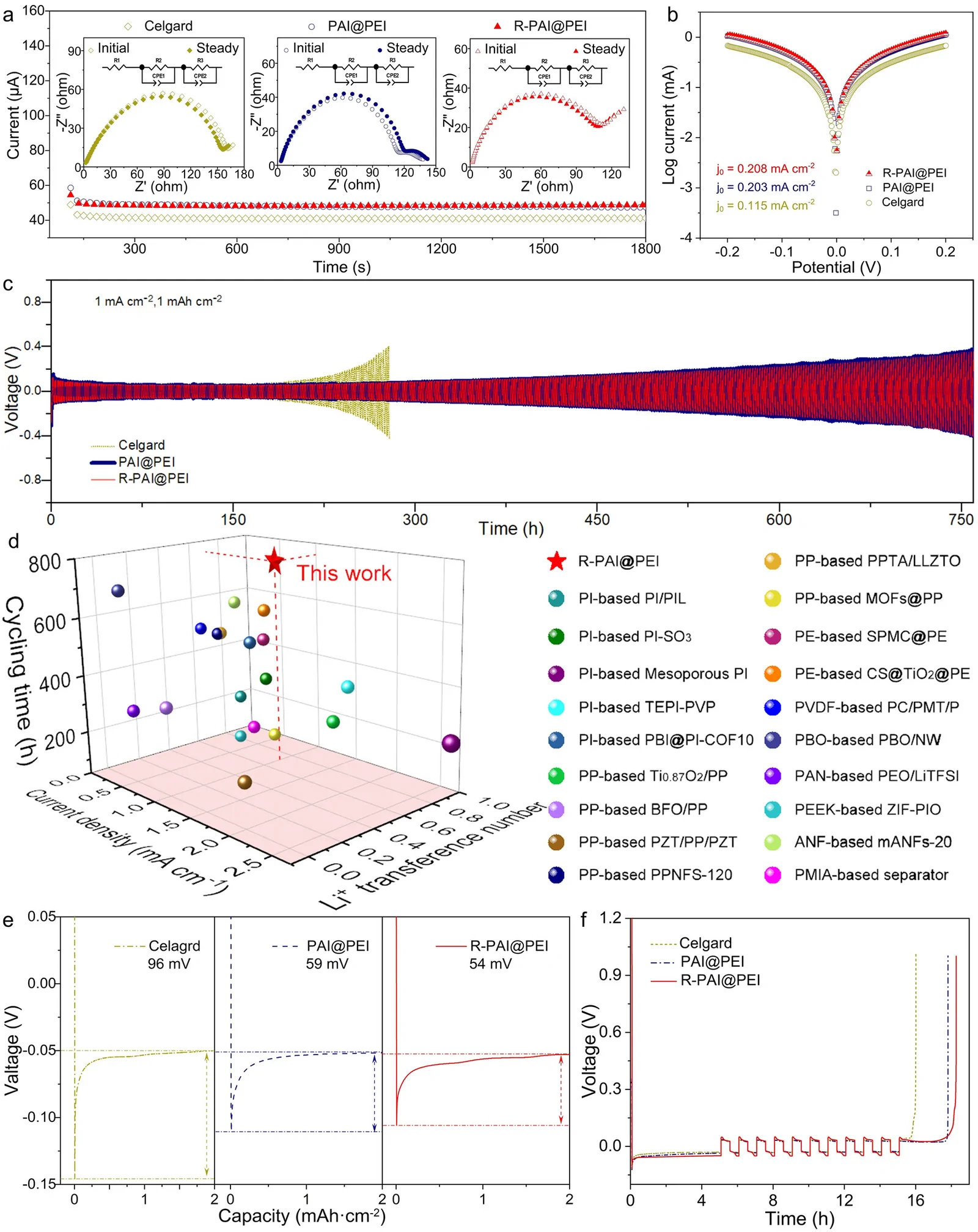
Li||Li and Li||Cu cells performance of the PAI@PEI-based separator. **a** Chronoamperometry plots for a Li||separator||Li cell. **b** Tafel plots for Li plating/stripping of the Li||Li cells. **c** Voltage − time curves for Li deposition/stripping process at 1 mA cm^−2^. **d** Comparison of *t*_Li_^+^ and cycling stability of PAI@PEI-based Li||Li cells with reported state-of-the-art separators. **e** Voltage profiles and **f** CE_avg_ of Cu||Li cells for the various separators

The lithium-ion transference number (*t*_Li_^+^) was determined using electrochemical impedance spectroscopy (EIS) on symmetric Li||Li cells with a potential polarization. The PAI@PEI and R-PAI@PEI separators exhibit higher *t*_Li_^+^ values of 0.66 and 0.71, respectively, compared to the polyolefin-based Cegard separator with a *t*_Li_^+^ value of 0.43 (Figs. 3a and S20). The polar PAI and PEI units of the separator facilitate Li^+^ desolvation via ion–dipole interactions between the imide/amide/ether groups and Li^+^, improving Li^+^ mobility. In contrast, the Celgard separator exhibits a slow Li^+^ migration because of its non-polar structure, which promotes the formation of larger solvation clusters within electrolyte. The large-scale solvated Li^+^ clusters can shuttle through the Cegard separator, resulting in reduced Li^+^ mobility, a strong interfacial electric field, and uneven Li^+^ deposition [65]. The polar amide and imide groups of the PAI@PEI separator provide additional active sites for Li^+^ adsorption, weakening Li^+^-solvent interaction and facilitating faster Li^+^ transport. The PAI@PEI and R-PAI@PEI separators demonstrate lower areal resistance than the Celgard separator (Fig. S21), reflecting their superior ionic conductivity of 1.60 and 1.63 mS cm^−1^, respectively. These values significantly surpass the Celgard’s conductivity, which is only 0.79 mS cm^−1^. The high ionic conductivity of PAI@PEI and R-PAI@PEI separators is primarily attributed to their high porosity, low tortuosity, enhanced wettability, and increased Li^+^ mobility, all of which are facilitated by the PAI and PEI matrix. This increased Li^+^ mobility also plays a crucial role in uniform Li deposition, thereby inhibiting the formation of Li dendrites. Further analysis of the impedance of Li||Li cells reveals the interfacial stability of the PAI@PEI separator (Fig. S22). After one cycle, cell with the Celgard separator shows an elevated interfacial resistance of approximately 124 Ω, compared to 60 and 64 Ω observed in R-PAI@PEI and PAI@PEI separators, respectively. After 10 cycles, the resistance for the Celgard decreases to around 70 Ω, while R-PAI@PEI and PAI@PEI separators maintain consistently lower resistances of 39 and 42 Ω, respectively. The lower resistance of the R-PAI@PEI and PAI@PEI separators indicates a more stable interface with the Li anode, contributing to enhanced cycling performance. The exchange current density (*i*_0_) of Li||Li cells was also calculated from the Tafel profiles, reflecting the kinetic resistance associated with the Li deposition/stripping process (Fig. 3b). The *i*_0_ values for R-PAI@PEI (0.21 mA cm^−2^) and PAI@PEI (0.20 mA cm^−2^) surpass that of Celgard (0.12 mA cm^−2^), suggesting a faster electrochemical reaction at the SEI/lithium interface.

To evaluate the cycling stability of these separators, galvanostatic cycling tests were conducted on Li||Li symmetric cells equipped with various separators (Fig. 3c). The Celgard-based Li||Li cell exhibits pronounced voltage fluctuations during Li stripping and deposition cycles, indicating the growth of Li dendrites due to the nonuniform ion flux. In contrast, the Li||Li cells with the R-PAI@PEI and PAI@PEI separators maintain consistent voltage profiles over 750 h at the current density of 1 mA cm^−2^, demonstrating effective suppression of dendrite growth. This is further supported by the smooth surface microstructure of the cycled Li metal in Li||Li cells using the R-PAI@PEI and PAI@PEI separators (Fig. S23). In contrast, cell using the Celgard separator displays rough Li metal surfaces. Simultaneously, PAI@PEI-based Li||Li cells separator also exhibits good cycling stability compared with the Celgard separator at the current density of 2 and 3 mA cm^−2^ (Fig. S24). Moreover, the PAI@PEI-based Li||Li cells also exhibit lower plating/stripping overpotentials than that of Celgard separator under various current densities (0.5–2 mA cm^−2^) (Fig. S25). Importantly, both the Li^+^ transference number and exceptional cycling stability at 1 mA cm^−2^ of R-PAI@PEI-based Li||Li cells significantly outperform those of modified PI-based, polyolefin-based, and heat-resistant separators (e.g., PVDF, PEEK, and PAN), as shown in Fig. 3d and Table S3. Critical current density is an important parameter for evaluating Li^+^ deposition kinetics. As shown in Fig. S26, the Li||Li cell with the Celgard membrane experiences a short circuit at a current density of 4.4 mA cm^−2^, while Li||Li cells with the PAI@PEI and R-PAI@PEI membranes reach 6.92 and 7.52 mA cm^−2^, respectively. This demonstrates that PAI@PEI-based separators can enhance Li^+^ transport kinetics through the electrode/electrolyte interfaces while facilitating the uniform Li^+^ distribution and Li dendrite suppression. To further assess the separator’s ability of inhibiting Li dendrite growth, the Cu||Li cells were assembled to measure nucleation overpotential (Fig. 3e). Cells with the R-PAI@PEI (~ 54 mV) and PAI@PEI separators (~ 59 mV) demonstrate lower nucleation overpotentials compared to cell with the Celgard (~ 96 mV) separator. This reduction in overpotential is directly linked to the suppression of irregular Li nucleation and enhanced uniformity in Li^+^ deposition. The electrochemical reversibility of Li deposition was quantitatively evaluated by analyzing the average coulombic efficiency (CE_avg_) in Li||Cu asymmetric cells. As shown in Fig. 3f, the cells using the PAI@PEI and R-PAI@PEI separators exhibit enhanced cycling stability, achieving a CE_avg_ of 77.2% and 82% over 10 cycles, surpassing the Celgard (67.7%) separator. The morphology of Li deposition in Li||Cu cells is shown in Fig. S27. The Celgard separator leads to significant mossy Li dendrite formation, while cells with PAI@PEI and R-PAI@PEI separators exhibit smooth, dense, and uniform Li deposition microstructures. The Cu foil surfaces reveal that R-PAI@PEI-based and PAI@PEI-based cells show no accumulation of dead Li, whereas substantial dead Li is present in Li||Cu cells with the Celgard separator (Fig. S28). The Sand’s time was investigated to verify the impact of separator for ion transport kinetics (Fig. S29). The voltage changes of Li||Cu cells with the Celgard, PAI@PEI, and R-PAI@PEI occur at 7.2, 13.4, and 17.0 h, respectively. The extended Sand’s time displays enhanced ion transport capability, suggesting that the PAI@PEI-based separator effectively improves Li^+^ flux and mitigates dendrite growth. These results highlight that the R-PAI@PEI separator, after recovering its apertures, still enables uniform Li^+^ transport, deposition, and stripping, effectively suppressing dendrite formation.

**Fig. 4:**
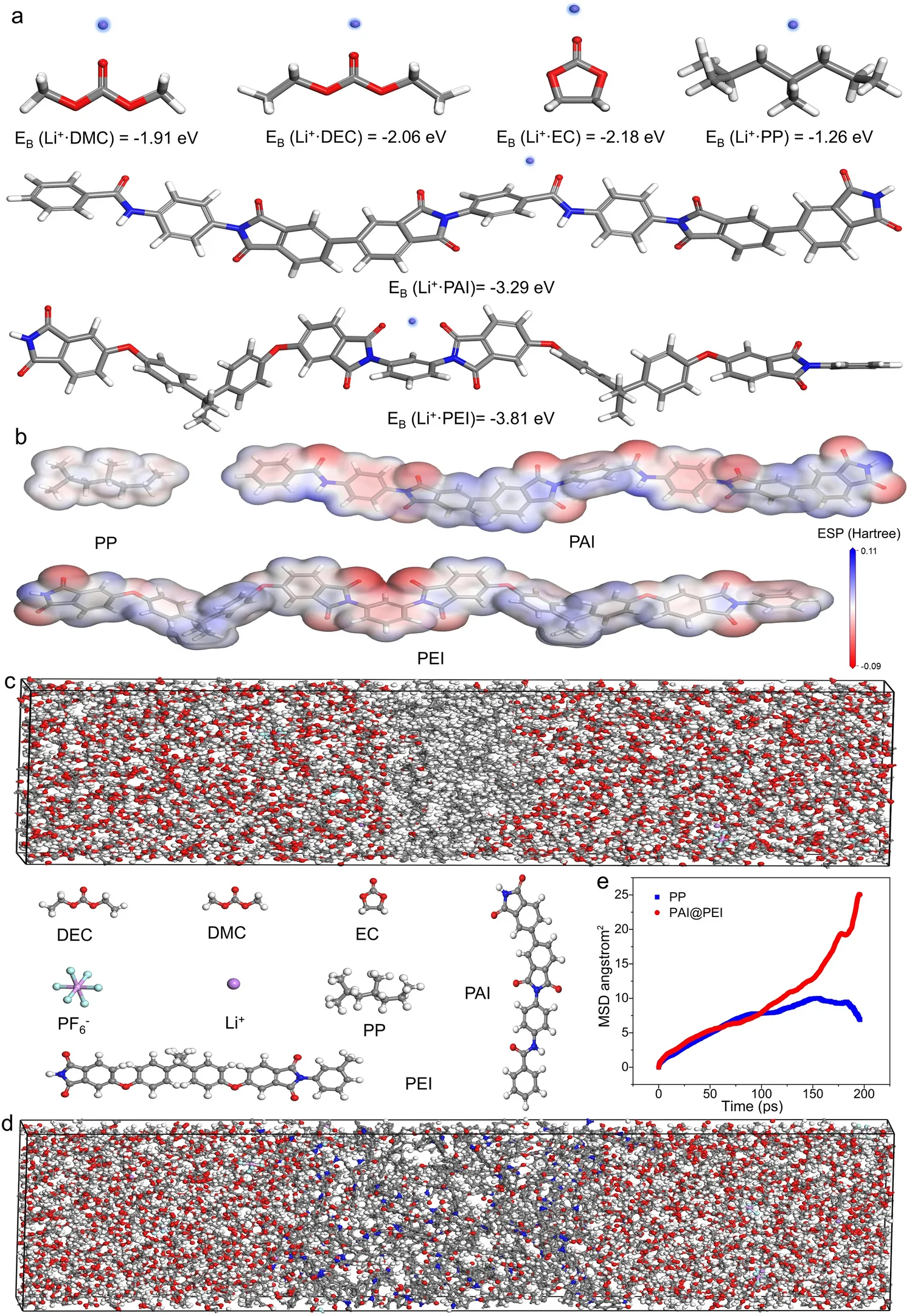
Interactions investigation. **a** E_B_ for Li^+^ with electrolytes, PP, PAI, and PEI. **b** Electrostatic potential maps of the PP, PAI, and PEI. The molecular dynamics (MD) boxes at equilibrated configurations of **c** PP and **d** PAI@PEI. **e** Calculated mean square displacements (MSDs) of PP and PAI@PEI

Density functional theory (DFT) calculations were conducted to further investigate the interaction between PAI@PEI and Li^+^, aiming to elucidate the mechanism of Li^+^ deposition. As shown in Fig. 4a, the binding energies (E_B_) of Li^+^ with PEI, PAI, polypropylene (PP), and various electrolytes (DEC, DMC, and EC) are evaluated and compared. The binding energies of PAI and PEI with Li^+^ are found to be notably higher, with values of − 3.29 and − 3.81 eV, respectively, significantly surpassing those of DEC (− 2.06 eV), DMC (− 1.91 eV), and EC (− 2.18 eV). Furthermore, the E_B_ of PAI and PEI also exceeds those of PP (− 1.26 eV), highlighting the enhanced affinity ability of PAI and PEI with Li^+^. The strong binding affinity provides abundant active sites for Li^+^ adsorption, facilitating uniform Li^+^ migration, in contrast to the Celgard separator, which lacks Li^+^ attracting functionality. Figure 4b shows the electrostatic potential, which can conjecture the functional sites for analyzing the electrostatic interaction between PAI/PEI molecule and Li^+^, in contrast to the non-polar polypropylene structure. The negative charges are predominantly localized on the amide, imide, and ether groups, which serves as the chelating sites for Li^+^ and further confirms the binding affinity of PAI and PEI with Li^+^. Thus, the strong binding affinity of PAI@PEI-based separator can facilitate Li^+^ dissociation and regulate Li^+^ uniform transport, effectively suppressing dendrite formation. Molecular dynamics (MD) simulations were employed to explore the Li^+^ transport behavior. Two box systems were constructed, both incorporating the same liquid electrolyte composition, but with different polymer layers: one using PP and the other employing the PAI@PEI polymer layer, as depicted in Fig. 4c, d. The electrolyte system in this simulation consists of 1 mol L^−1^ LiPF_6_, with the solvent being a 1:1:1 mixture of EC, DEC, and DMC, consistent with the experimental setup. The mean square displacement (MSD) of Li^+^ was then calculated to evaluate diffusion behavior. The MSD, defined as the deviation of a particle’s position from its reference point over time, serves as a measure of the Li^+^ diffusion rate. The MSD curves for the different separators are shown in Fig. 4e. Notably, the slope of the MSD for Li^+^ is twice as steep in the PAI@PEI separator + liquid electrolyte (0.097) compared to the PP separator + liquid electrolyte (0.043), indicating a significantly higher migration efficiency of Li^+^ in the PAI@PEI system. These results further underscore the critical role of the PAI@PEI separator in enhancing Li^+^ conductivity and promoting rapid ion transport.

**Fig. 5:**
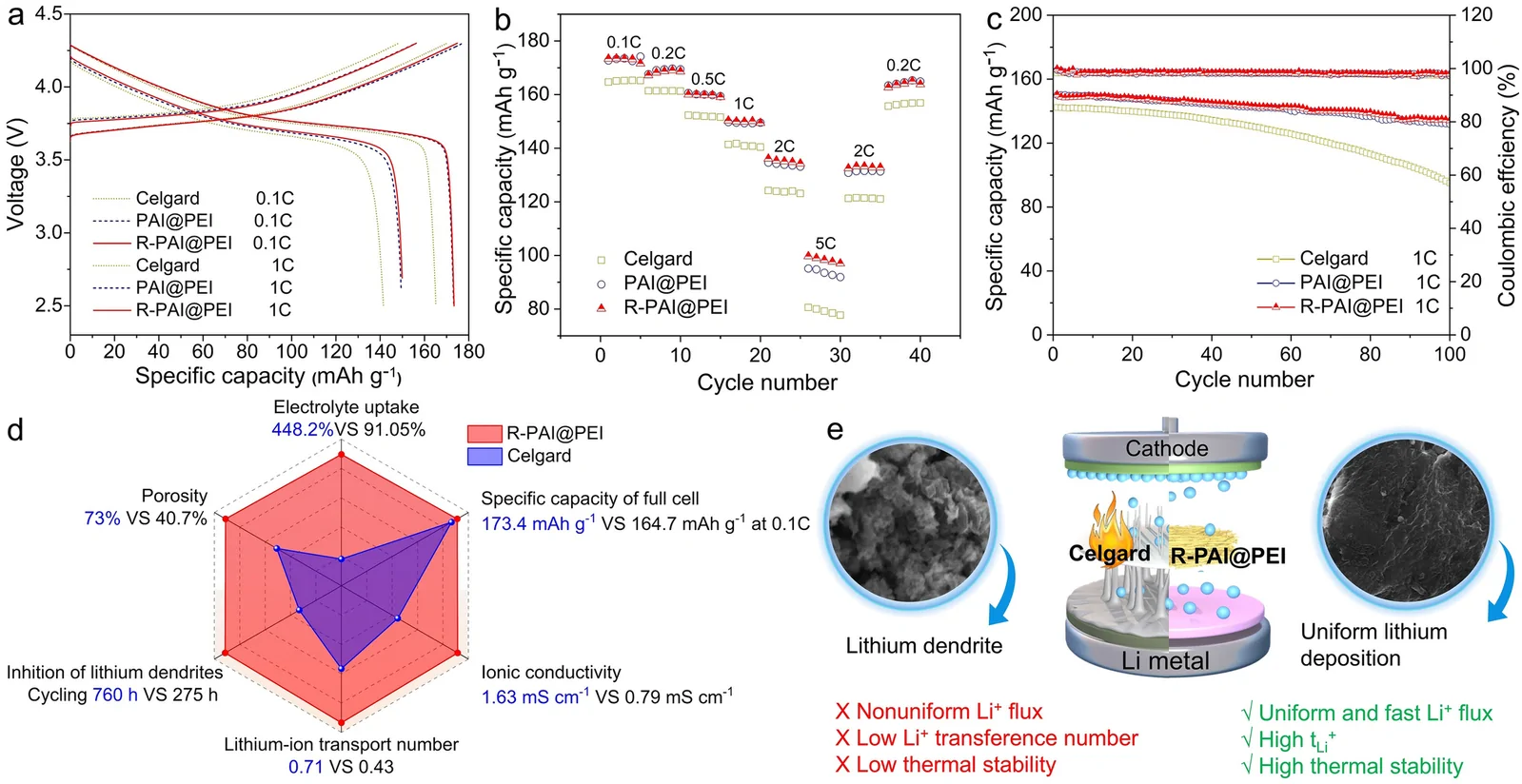
Full-cell evaluations and performance advantage of the R-PAI@PEI separator. **a** Charge–discharge profiles, **b** rate capability, **c** cycling stability of the Li||NCM523 cells. **d** Radar plot comparing the Celgard and R-PAI@PEI separators. **e** Schematics comparing separators performance in cells

To comprehensively evaluate the practical applicability of separators in LMBs, the electrochemical performance of Li||NCM523 full cells was investigated. The EIS of Li||NCM523 cells using R-PAI@PEI and PAI@PEI separators exhibit charge-transfer resistances of 101 and 108 Ω, respectively, both of which are markedly lower than that of the Celgard-based cell (157 Ω) (Fig. S30). This reduction in resistance can be attributed to the abundant ion transport pathways and sufficient electrolyte uptake. Such structural advantages can enhance interfacial wettability and accelerate electrochemical kinetics, thereby contributing to the superior electrochemical performance. Figure 5a shows the initial discharge profiles of Li||NCM523 cells equipped with different separators. Cells equipped with PAI@PEI and R-PAI@PEI separators demonstrate superior specific capacities of 173.3 and 173.4 mAh g^−1^ at 0.1C, respectively, and 149.4 and 149.9 mAh g^−1^ at 1C, respectively, outperforming those with the Celgard separator (165.1 and 141.5 mAh g^−1^). Moreover, the rate performance of Li||NCM523 cells also was investigated. The discharge capacities for cells with PAI@PEI, R-PAI@PEI, and Celgard separators are 172.7, 173.4, and 164.7 mAh g^−1^ at 0.1C, respectively (Fig. 5b), further validating the practicability and advancement of PAI@PEI before and after aperture restoration. Notably, during high-rate cycling tests from 0.1C to 5C, the PAI@PEI and R-PAI@PEI separators maintain high capacities of 95.1 and 99.7 mAh g^−1^ at 5C, exceeding the Celgard-based Li||NCM523 cells. Furthermore, upon returning to lower discharge rates of 1C, 0.5C, and 0.2C, the Li||NCM523 cells demonstrate substantial capacity recovery, indicating robust rate capability. After 100 cycles at 1C (Fig. 5c), the Li||NCM523 cells with the PAI@PEI and R-PAI@PEI separators exhibit excellent capacity retention of 87.9% and 90.0%, surpassing that of the Celgard (66.8%) separator-based cell. SEM images (Fig. S31) reveal that Li metal surfaces in cells with the PAI@PEI and R-PAI@PEI separators remain smooth and compact morphology after cycling test, while cell with the Celgard separator exhibits pronounced dendritic formations. The X-ray photoelectron spectroscopy (XPS) verifies the electrochemical stability of the R-PAI@PEI separator during long-term cycling of Li||NCM523 cells (Fig. S32). Overall, the R-PAI@PEI separator represents a significant advancement in overall performance compared to the Celgard (Fig. 5d). Importantly, the electrochemical performance of the R-PAI@PEI separator is superior to that of the PAI@PEI separator owing to the smaller and more uniform pore size of the R-PAI@PEI separator. Figure 5e illustrates the Li^+^ deposition behavior of the R-PAI@PEI and commercial separators. The straight through-hole structure of the flammable Celgard separator causes uneven Li^+^ distribution on the Li anode surface, promoting dendritic Li growth that may puncture the separator and lead to safety risks. In contrast, the smart R-PAI@PEI can effectively diffuse Li^+^ through its polar groups and 3D nanofiber network, which can inhibit dendrite growth. These results demonstrate that the R-PAI@PEI separator effectively mitigates dendrite formation and presents the remarkable specific capability and capacity retention, highlighting its potential for next-generation high-safety LMBs.

## Conclusions

In summary, we have engineered a core–shell PAI@PEI separator, where the PEI core enables the separator to close its aperture structure at 400 °C, while the PAI shell facilitates the recovery of the apertures through thermal triggering at 350 °C. This structure imparts exceptional properties to the PAI@PEI-based separator, including high ionic conductivity (1.63 mS cm^−1^), satisfying Li⁺ transference number (0.71), a reduced nucleation overpotential (54 mV), and an elevated average coulombic efficiency (82%). The DFT calculation results verify that PAI@PEI can dissociate the Li^+^ and PF_6_^−^ from electrolytes, which can be adsorbed near the affinity sites of PAI and PEI, thereby enabling the PAI@PEI-based separator to restrain the growth of Li dendrites. Therefore, Li||Li symmetric cells with the PAI@PEI-based separator demonstrate minimal voltage polarization during Li plating/stripping cycles over 750 h. Furthermore, Li||NCM523 full cells retain 90.0% of their initial capacity after 100 cycles and deliver 99.7 mAh g^−1^ at a 5C rate. This work provides valuable insights for designing high-safety functional separators that can meet the demands of other energy-storage devices requiring safe and controlled energy delivery.

## Supplementary Information

Below is the link to the electronic supplementary material.

Supplementary file1 (DOCX 38730 KB)
