# Supplementary material for: Thermal-Gated Self-Repairing Polyimide Separator for Dendrite-Suppressed Lithium Metal Batteries
Source: Nanomicro Lett. 2026 Jan 30;18:228. doi: 10.1007/s40820-025-02050-2 (PMC12855706; doi:10.1007/s40820-025-02050-2)
Supplement: Supplementary file 1 — Supplementary file1 (DOCX 38730 KB) [file 40820_2025_2050_MOESM1_ESM.docx]

Supporting Information for

**Thermal-Gated Self-Reparing Polyimide Separator for Dendrite-Suppressed Lithium Metal Batteries**

Pengpeng Li^1+^, Xinluo Li^1+^, Yisong Zhou^1^, Yingying Zhang^1^, Nianyu Yue^1^, Jiameng Li^1^, Yumeng Xin^1^, Lianlong Hou^1^, Jiaji Yue^1^, Xin Zhang^1^, Guohua Sun^1, 3^* and Nanjun Chen^2^*

^1^ Hebei Key Laboratory of Flexible Functional Materials, College of Materials Science and Engineering, Hebei University of Science and Technology, Shijiazhuang 050018, P. R. China

^2^ State Key Laboratory of Physical Chemistry of Solid Surfaces, College of Chemistry and Chemical Engineering, Xiamen University, Xiamen, 361005, P. R. China

^3^ State Key Laboratory of Organic-Inorganic Composites, Beijing University of Chemical Technology, Beijing 100029, P. R. China

*^+^* Pengpeng Li and Xinluo Li contributed equally to this work.

*Corresponding authors. E-mail: [sungh@hebust.edu.cn](mailto:sungh@hebust.edu.cn) (Guohua Sun); [nanjun.chen@xmu.edu.cn](mailto:nanjun.chen@xmu.edu.cn) (Nanjun Chen)

**S1 Experimental Section**

**S1.1 Materials**

The following chemicals were utilized in this study: 3,3’,4,4’-biphenyl tetracarboxylic dianhydride (BPDA), polyetherimide (PEI), and 4,4’-diamino benzanilide (DABA), all of which were procured from Energy Chemical. Carbonate-based electrolytes, including dimethyl carbonate (DMC), diethyl carbonate (DEC), and ethylene carbonate (EC), along with N, N-dimethylacetamide (DMAc), 1-methyl-2-pyrrolidinone (NMP), and auxiliary reagents, were acquired from Suzhou Duoduo Chemical Technology Co., Ltd. Additionally, LiNi_0.5_Co_0.2_Mn_0.3_O_2_ (NCM523) cathodes were sourced from Guangdong Candlelight New Energy Technology Co., Ltd. The Celgard-2400 separator, known as Celgard, was used for comparison.

**S1.2 Porosity measurements**

Porosity was determined by calculating the mass difference of the separator before and after immersion in *n*-butanol, as shown in Eq. (S1):

$$\begin{aligned} \text{Porosity=}\frac{\left( {\text{m}_{\text{b}}}/{\text{ρ}_{\text{b}}} \right)}{\left( {\text{m}_{\text{b}}}/{\text{ρ}_{\text{b}}}\text{+}{\text{m}_{\text{p}}}/{\text{ρ}_{\text{p}}} \right)}\text{×100\%}\#\text{(}\text{S}\text{1)} \end{aligned}$$

where m_p_ and m_b_ represent the masses of the dried membrane and *n*-butanol, respectively, while ρ_b_ and ρ_p_ are the densities of *n*-butanol and separator.

**S1.3 Electrolyte uptake measurements**

To determine the electrolyte uptake, the mass difference of the separators before (W_2_) and after (W_1_) immersion in a ternary electrolyte (EC/DMC/DEC, 1:1:1 vol%) was measured. The electrolyte uptake was then calculated using Eq. (S2):

$$\begin{aligned} \text{Uptake=}\frac{\left( \text{W}_{\text{1}}\text{-}\text{W}_{\text{2}} \right)}{\text{W}_{\text{2}}}\text{×100\%}\#\text{(}\text{S}\text{2)} \end{aligned}$$

**S1.4 Ionic conductivity measurements**

The ionic conductivity of the separators was measured by recording the impedance of an SS||separator||SS cell with a CHI660 workstation. The conductivity was then calculated using Eq. (3):

$$\begin{aligned} \text{σ}\text{=}\text{d}/\left( \text{R}_{\text{d}}\text{∙S} \right)\#\left( \text{S}\text{3} \right) \end{aligned}$$

where d represents the membrane thickness, R_d_ is the bulk resistance, and S denotes the area between the separators and the stainless steel (SS) electrodes.

**S2 Density Functional Theory (DFT) Calculations**

Density functional theory (DFT) calculations were performed using the Gaussian software package [S1]. The objective was to evaluate the binding energies (E_B_) between Li⁺ ions and the repeat structural units of the electrolyte components. The generalized gradient approximation (GGA) with the Perdew–Burke–Ernzerhof (PBE) exchange–correlation functional was employed, as it offers a good balance between accuracy and computational cost in polymer–ion interaction studies. The 6-31G (d,p) basis set was applied for all atoms. Full geometry optimizations were performed without symmetry constraints at the same level of theory. Vibrational frequency analyses were subsequently conducted to verify that the optimized structures correspond to true minima on the potential energy surface (no imaginary frequencies) and to obtain zero-point energy (ZPE) and thermal corrections. The binding energy (E_B_) between Li⁺ and the polymer repeat structure was calculated using Eq. (S4):

$$\begin{aligned} \text{E}_{\text{B}}\text{=}\text{E}_{\text{complex}}\text{-}\text{E}_{\text{unit}}\text{-}\text{E}_{\text{ion}}\#\left( \text{S}\text{4} \right) \end{aligned}$$

where E_complex_, E_unit_, and E_ion_ are total energy, energy of optimized unit, and energy of optimized Li^+^.

**S3 Molecular dynamics (MD) simulations**

In this system, periodic boundary conditions were applied in all three directions. MD simulations were conducted using the GROMACS software package [S2]. The initial structure was first subjected to energy minimization using the steepest descent algorithm until the maximum force fell below 200 kJ·mol^-1^·nm^-1^. The CHARMM36 all-atom force field was employed due to its wide applicability to polymer and electrolyte systems [S3]. Long-range electrostatics were treated using the particle–mesh Ewald (PME) method, and van der Waals interactions were truncated at 1.2 nm [S4]. Following minimization, a 100 ps NPT ensemble MD simulation was performed at 298 K and 1 bar to relax the system volume. Temperature and pressure were controlled using the V-rescale thermostat and the Parrinello–Rahman barostat [S5, S6]. Subsequently, a 200 ps production run was conducted under the NVT ensemble at 298 K using the same thermostat. A time step of 1 fs was used for all simulations, and all covalent bonds involving hydrogen atoms were constrained using the LINCS algorithm. To investigate ion migration and field-induced structural evolution, a uniform external electric field was applied along the z-axis during the production MD simulation. Similar approaches have been previously adopted to study charge transport and polarization phenomena in polymer electrolytes and battery materials [S7].

**S4 Supplementary Figures and Tables**





**Fig. S1** Synthetic procedure of PAI


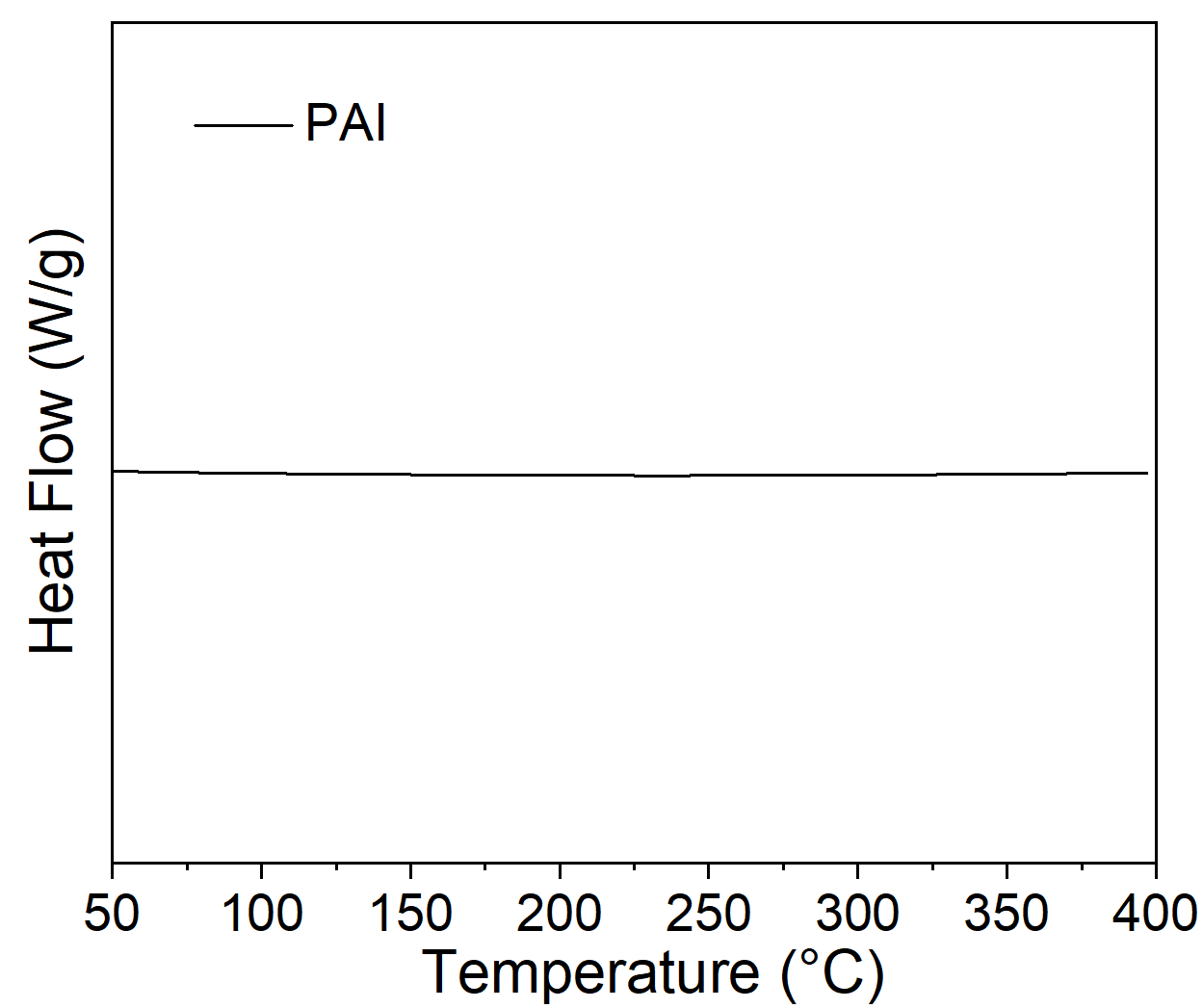


**Fig. S2** DSC curve of as-prepared PAI


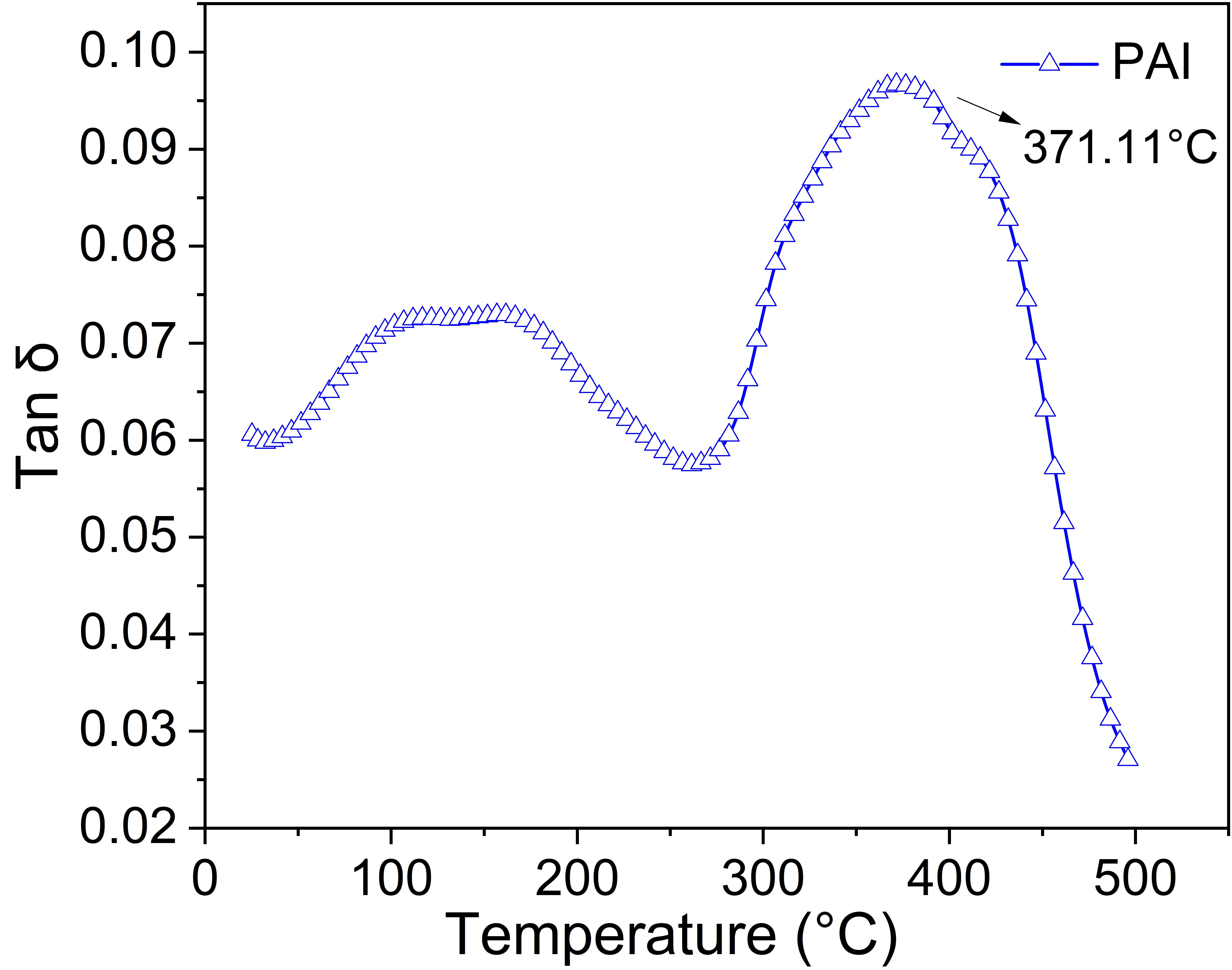


**Fig. S3** DMA curve of PAI film at 5 ℃/min with N_2_


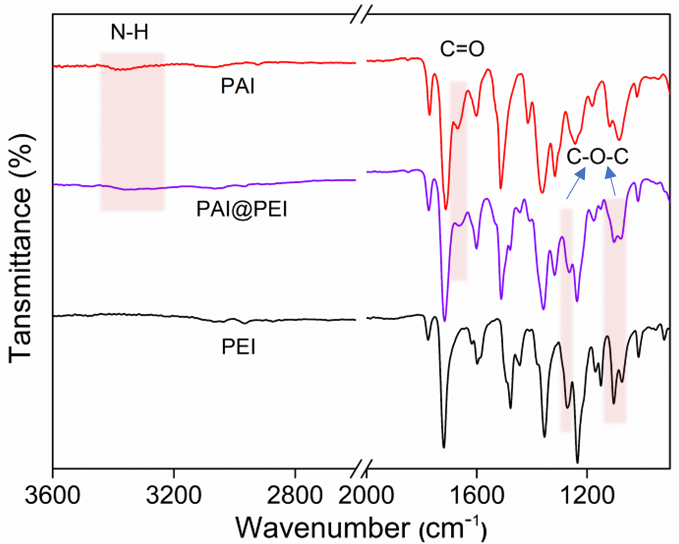


**Fig. S4** FTIR spectra of the PAI@PEI, PAI, and PEI samples


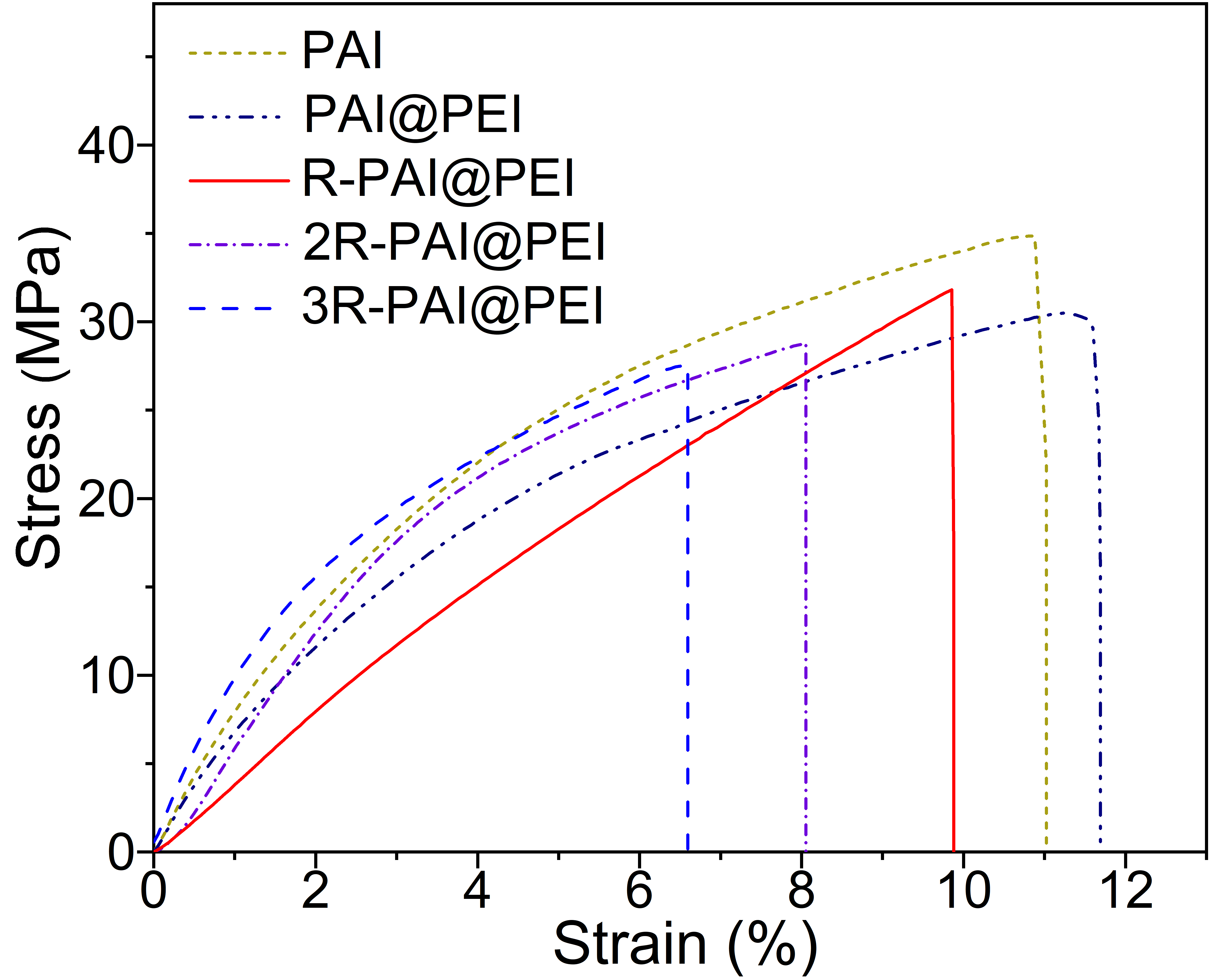


**Fig. S5** Mechanical properties of the PAI@PEI and PAI as well as PAI@PEI membranes with the first, second and third restored apertures (R-PAI@PEI, 2R-PAI@PEI, and 3R-PAI@PEI)

**
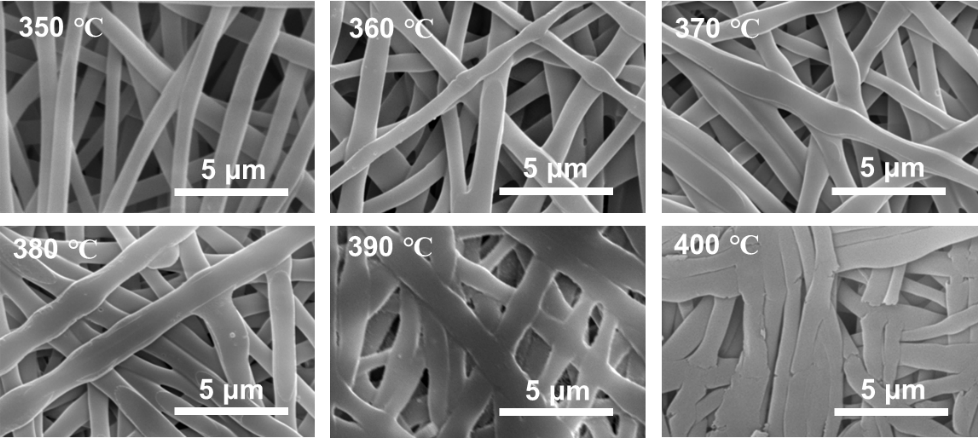
**

**Fig. S6** The surface morphology of PAI@PEI membrane at different temperatures


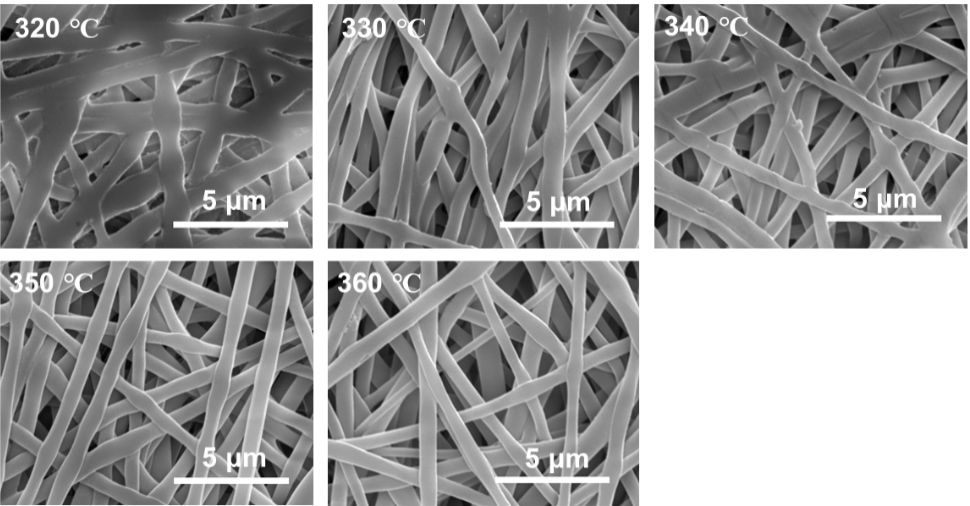


**Fig. S****7** The surface morphology of closed-aperture PAI@PEI membranes at different temperatures


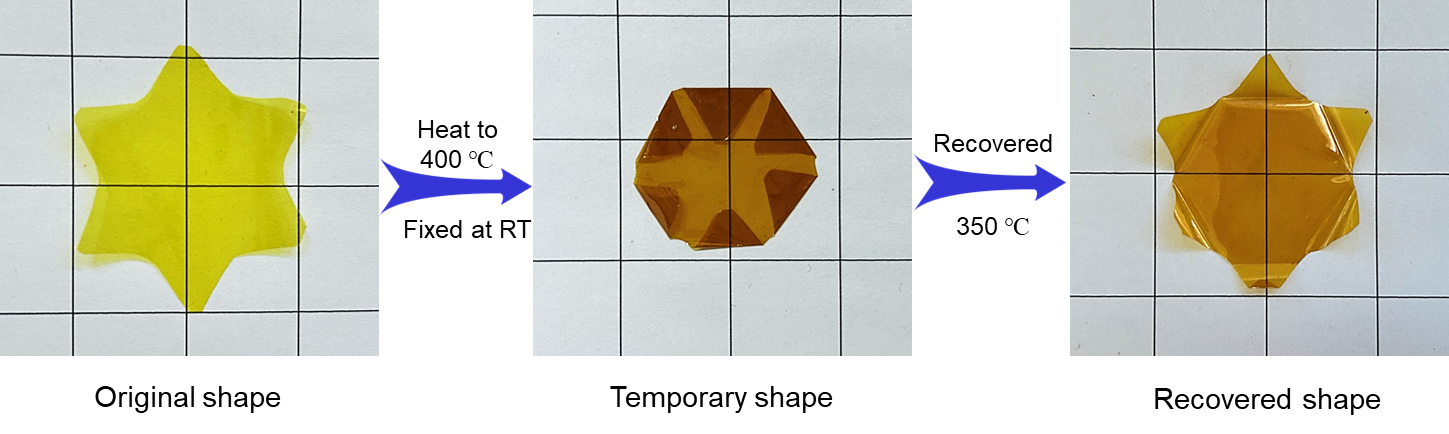


**Fig. S8** The entire shape memory process of a star-shaped film (original shape) at a high-temperature


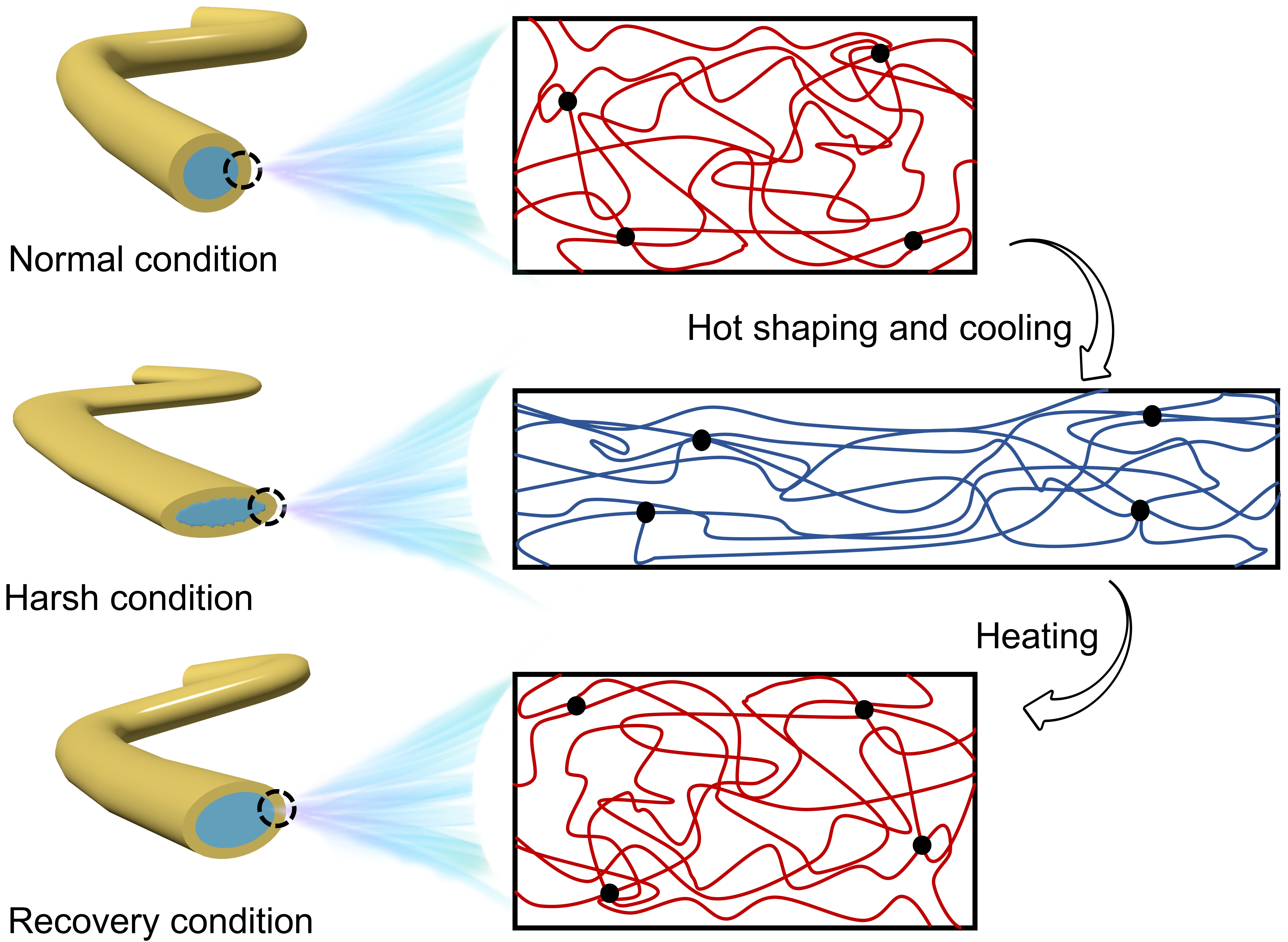


**Fig. S9** The schematic diagram of the self-recovering mechanism for PAI@PEI membrane





**Fig. S10** The fiber diameters of **a** PAI@PEI, **b** closed-aperture PAI@PEI (C-PAI@PEI), and **c** R-PAI@PEI; The aperture diameters of **d** PAI@PEI and **e** R-PAI@PEI membranes


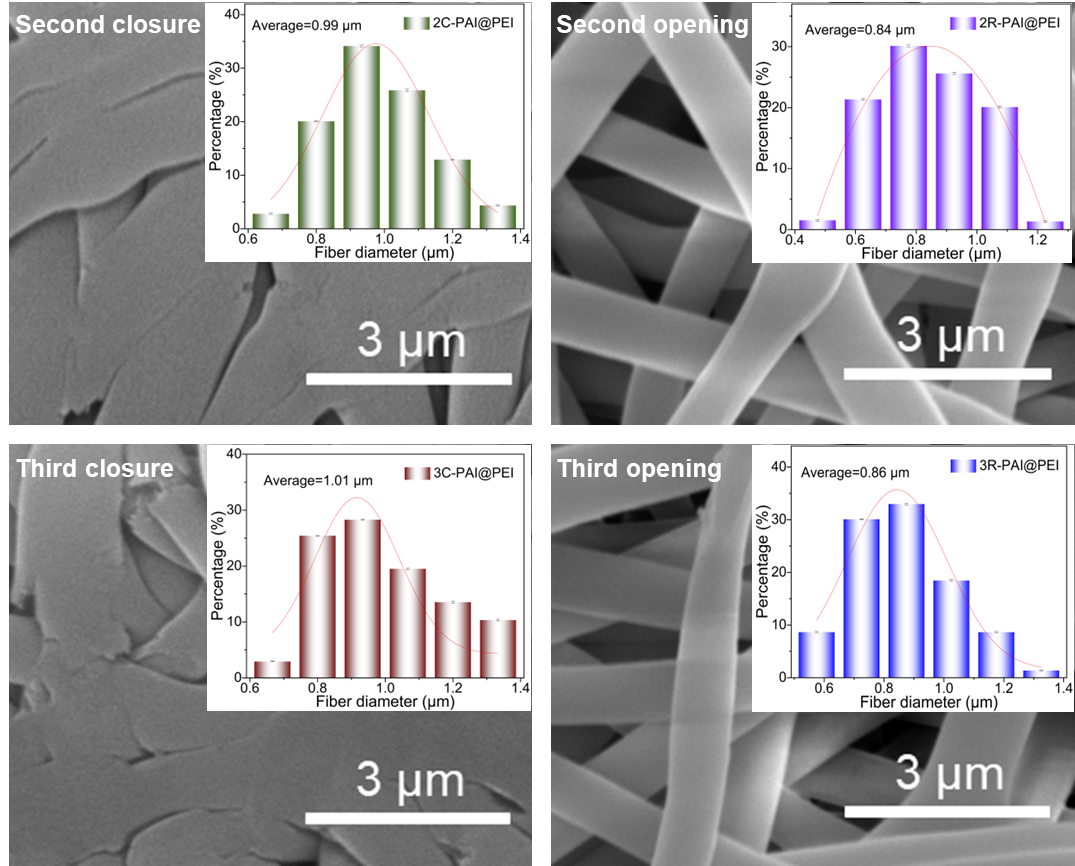


**Fig. S11** The surface morphology of the PAI@PEI membranes after repeated heating-cooling cycles


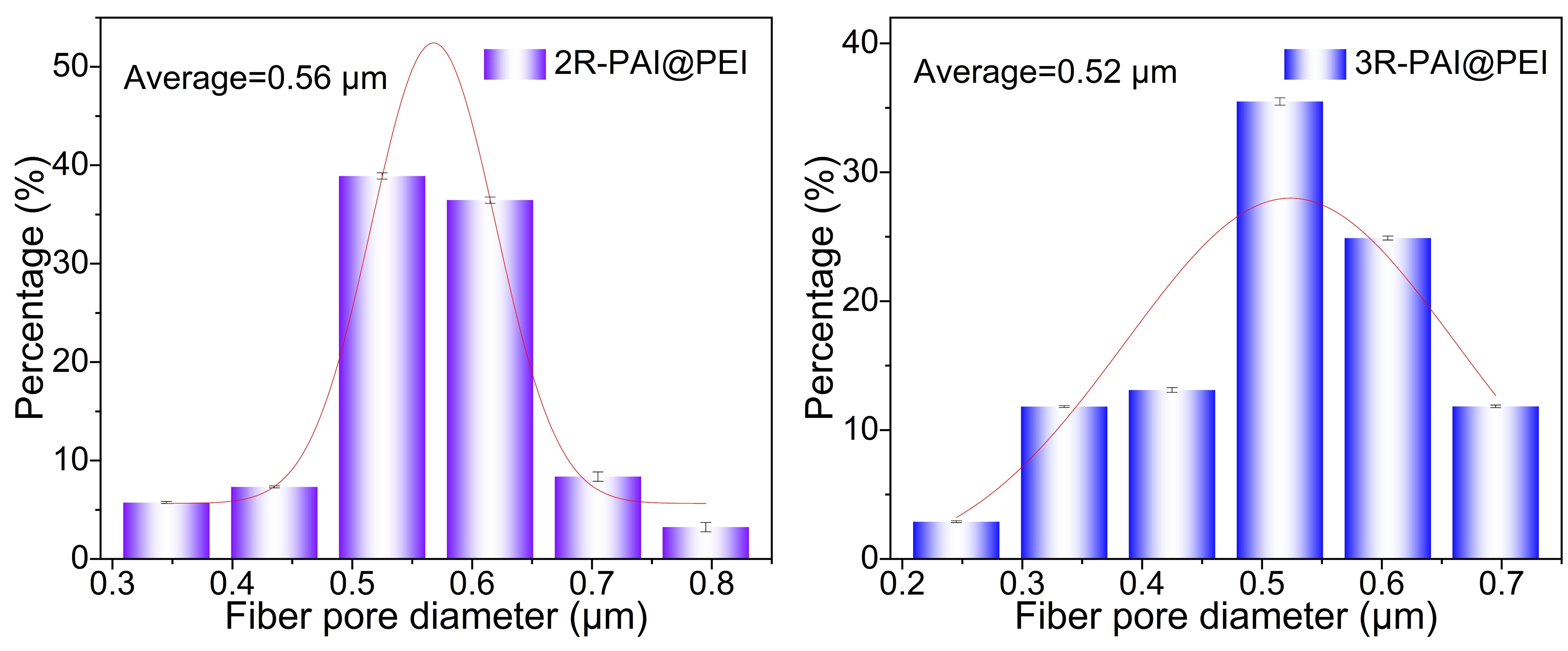


**Fig. S12** The aperture diameters of PAI@PEI membranes with the second and third restored apertures (2R-PAI@PEI and 3R-PAI@PEI)

**
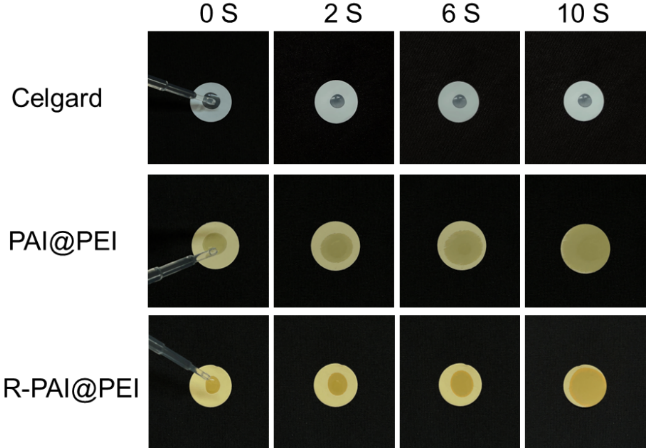
**

**Fig. S13** Photographs of wetting behavior of electrolytes on the Celgard, PAI@PEI, and R-PAI@PEI separators


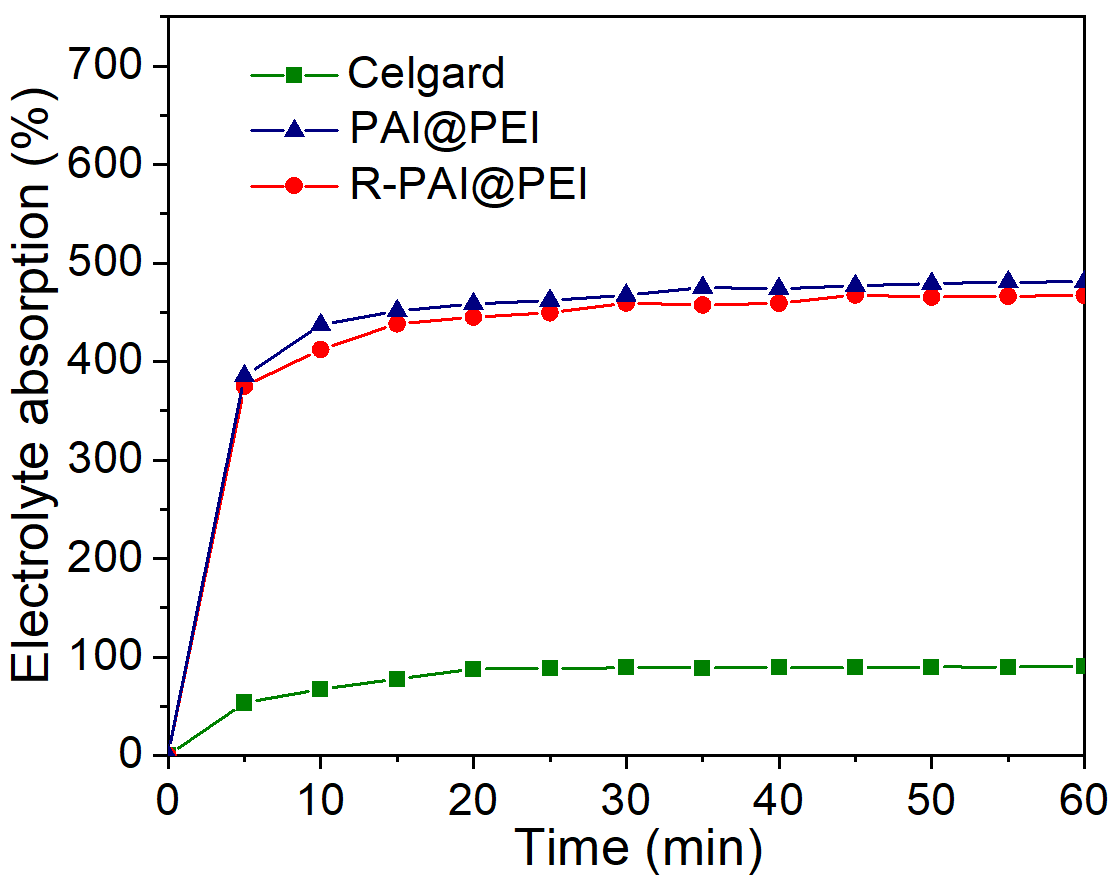


**Fig. S14** Electrolyte absorption and retention of the Celgard, PAI@PEI, and R-PAI@PEI samples


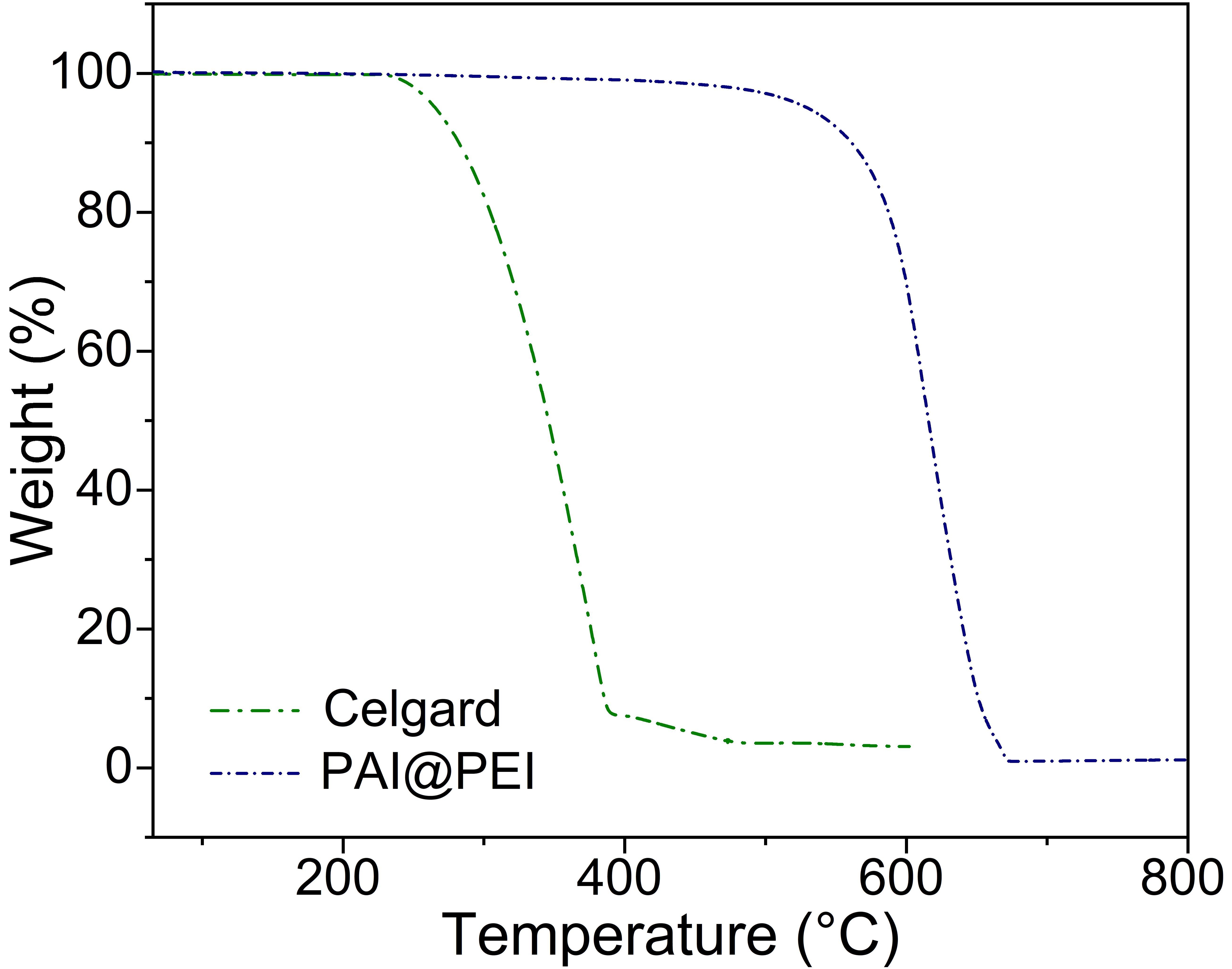


**Fig. S15** TGA curves of the PAI@PEI and Celgard membranes


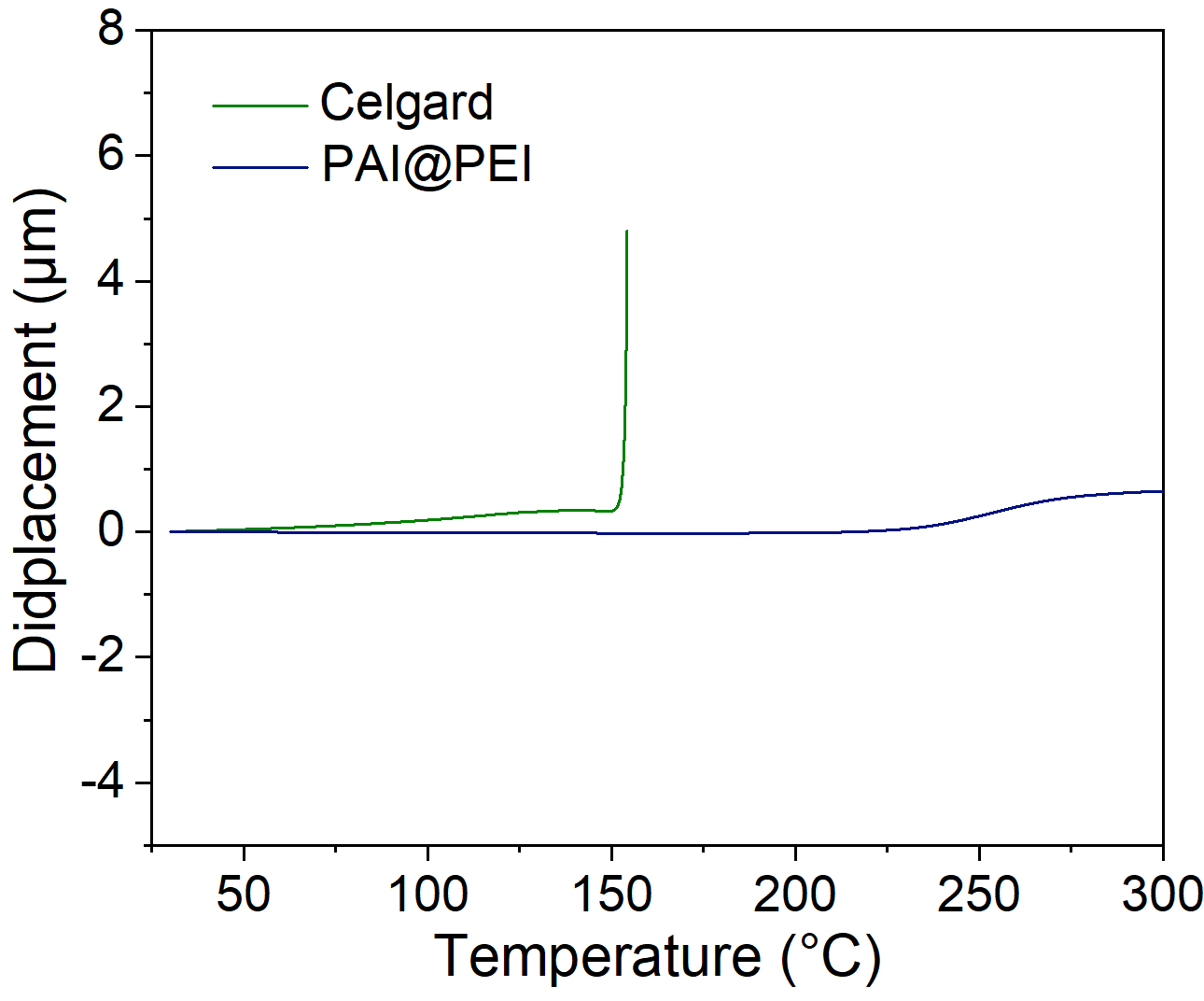


**Fig. S16** TMA curves of the PAI@PEI and Celgard samples


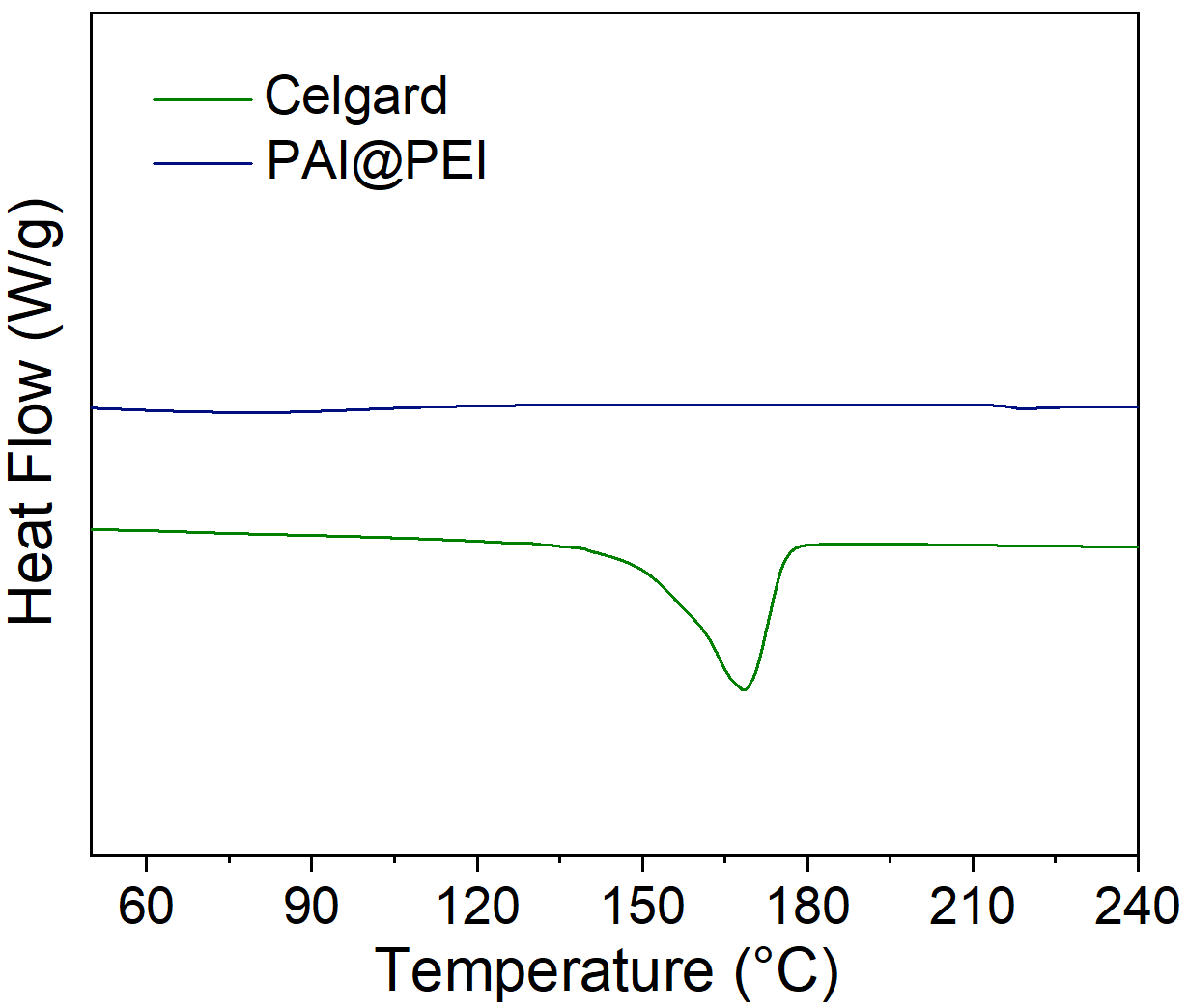


**Fig. S17** DSC curves of the PAI@PEI and Celgard samples


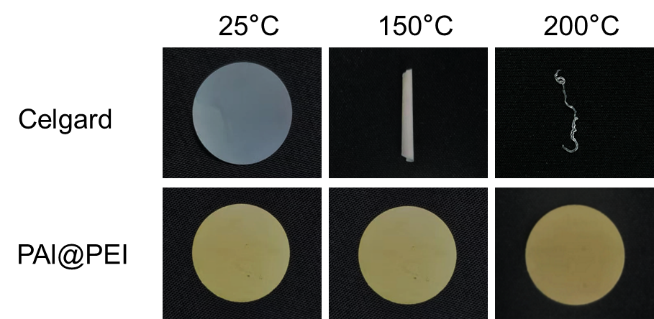


**Fig. S18** Digital photos showing thermal-dimensional stability of separators


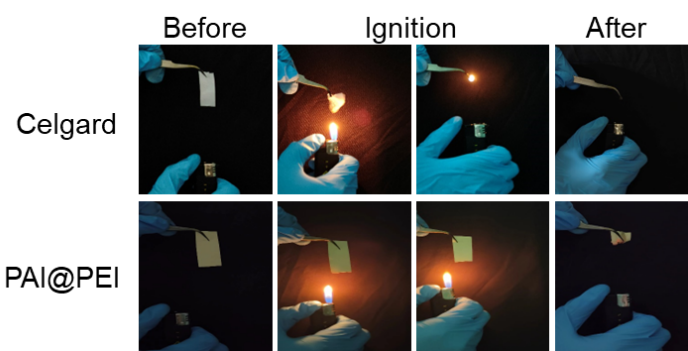


**Fig. S19** Ignition testing of the Celgard and PAI@PEI samples


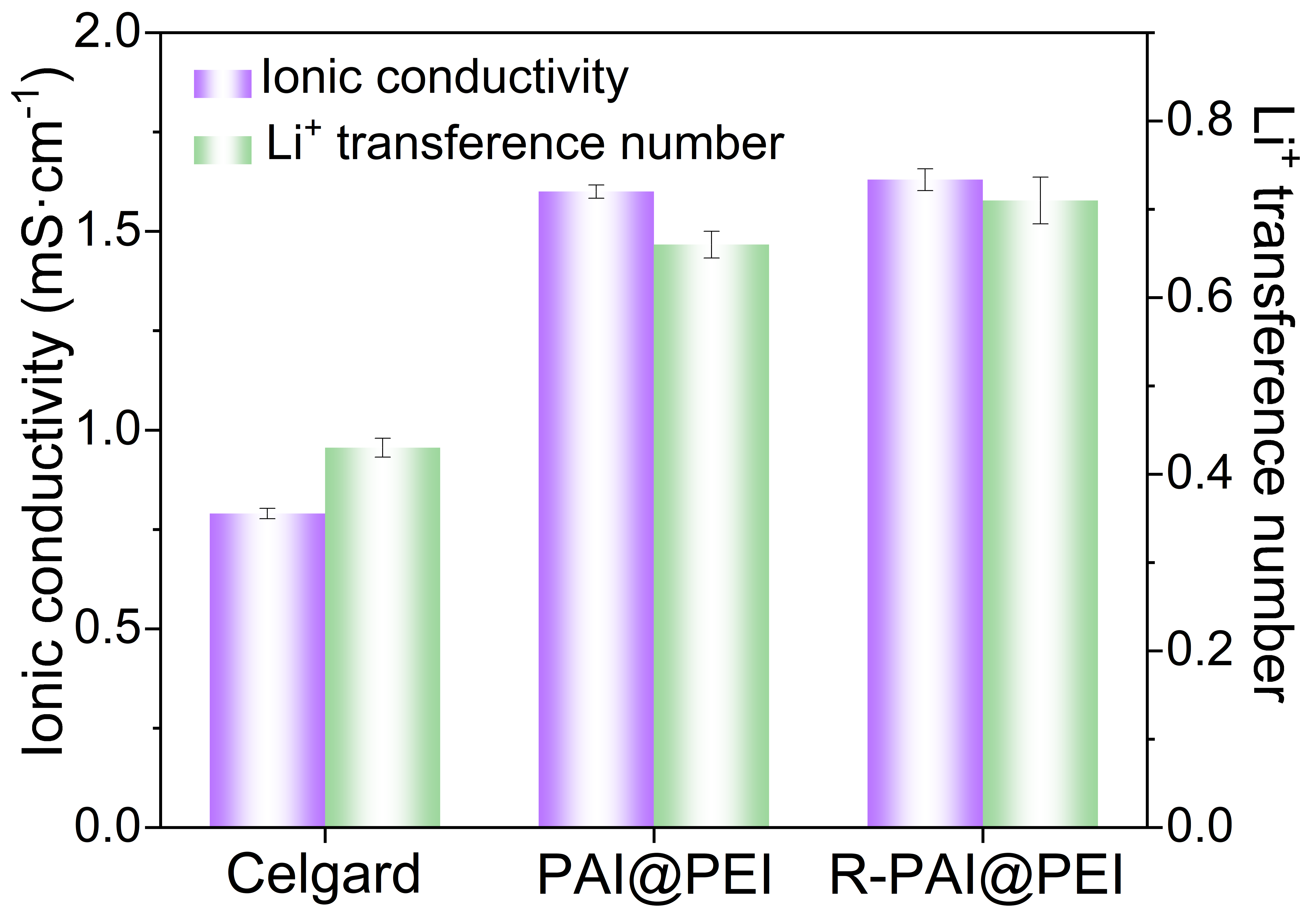


**Fig. S20** Comparison of the ionic conductivity and Li^+^ transference number


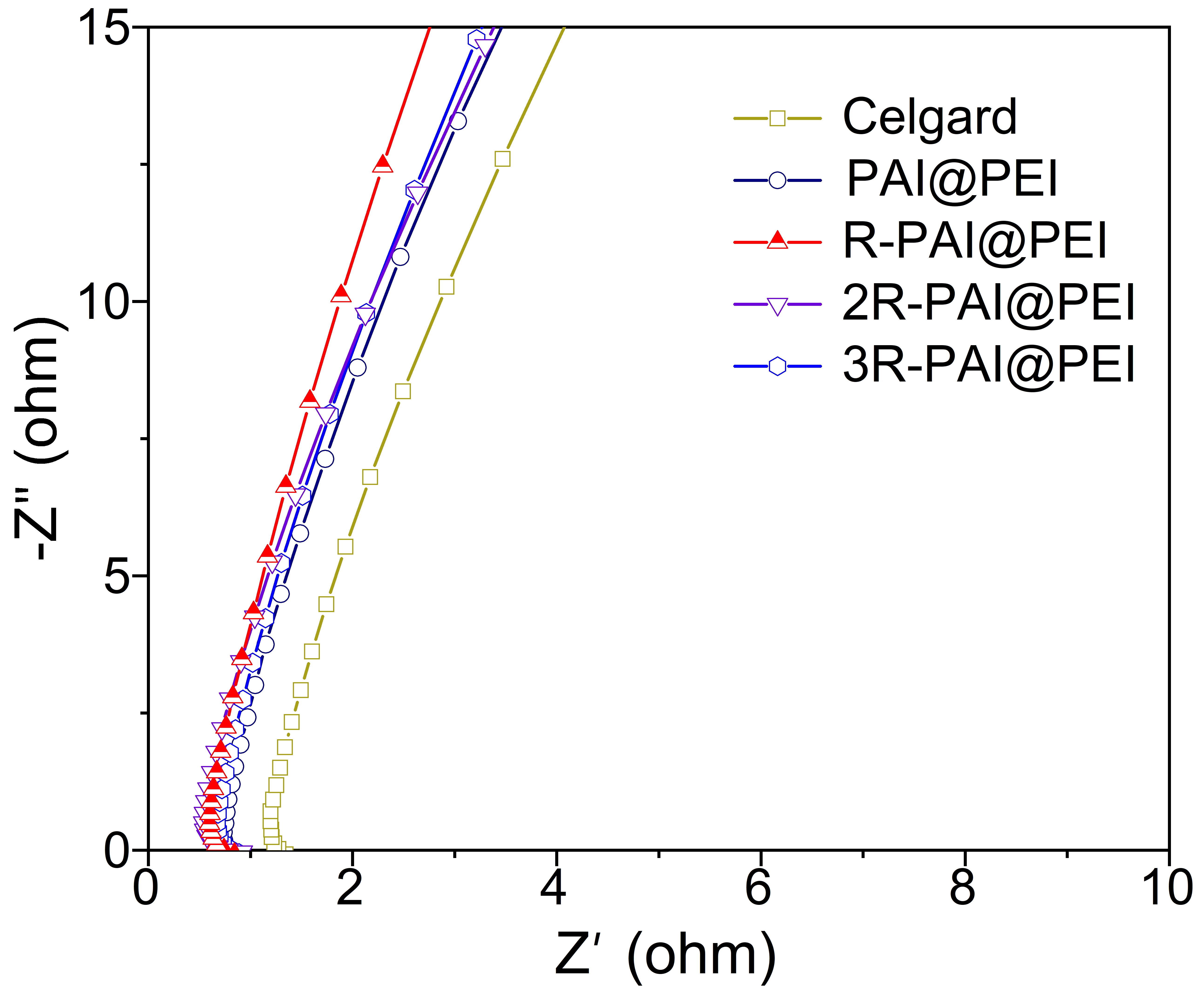


**Fig. S21** Nyquist plots of SS||SS batteries with the Celgard, PAI@PEI, R-PAI@PEI, 2R-PAI@PEI, and 3R-PAI@PEI separators


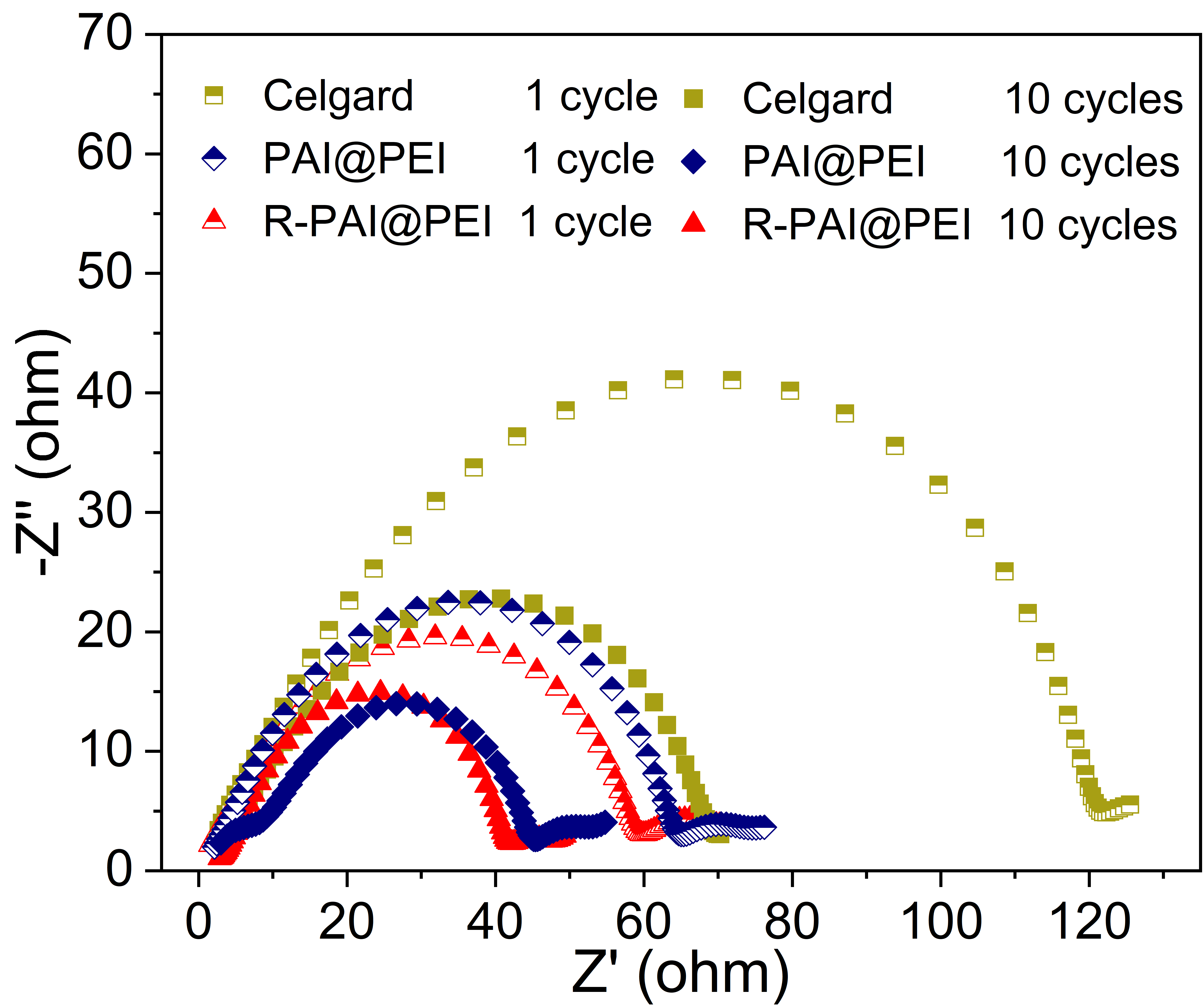


**Fig. S22** Nyquist plot of the impedance spectra of the Li||Li cells at 1 mA cm^-2^


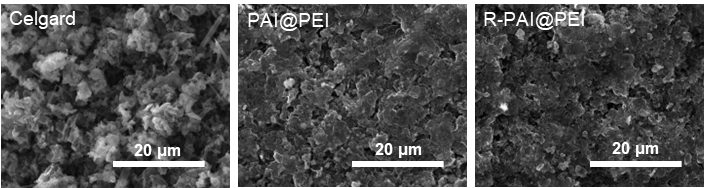


**Fig. S23** SEM images of Li anode of Li symmetrical cells using the Celgard, PAI@PEI, and R-PAI@PEI separators


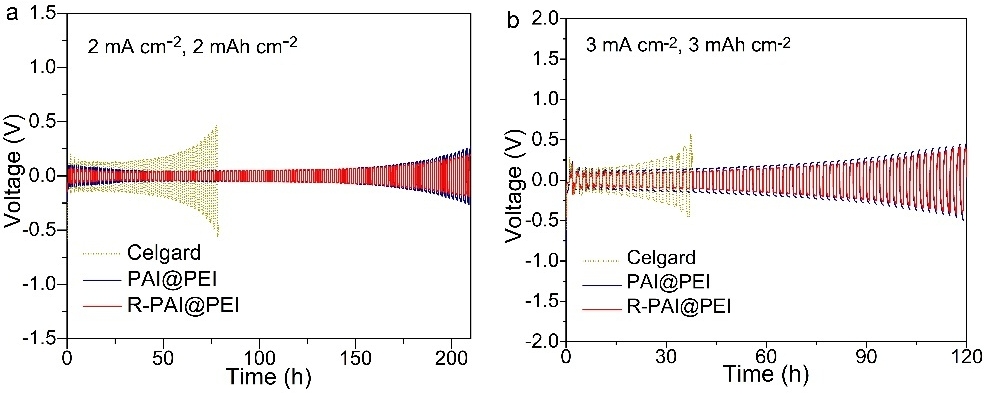


**Fig. S24** Cycling plating/stripping process of Li||Li cell at high current density of 2 and 3 mA cm⁻²


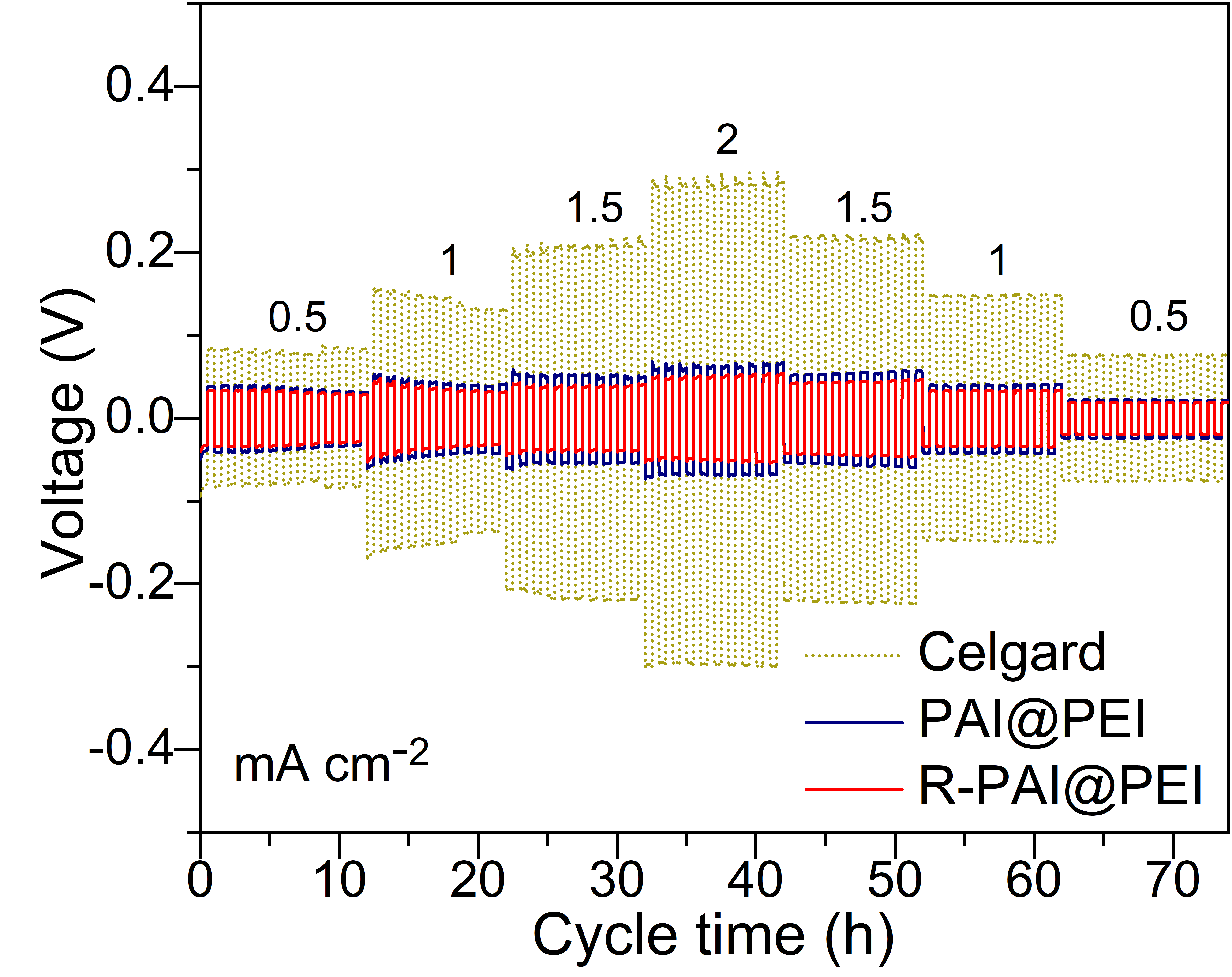


**Fig. S25** Rate performance at current density from 0.5 to 2 mA cm^−2^ with charge/discharge durations of 30 min in Li||Li cells


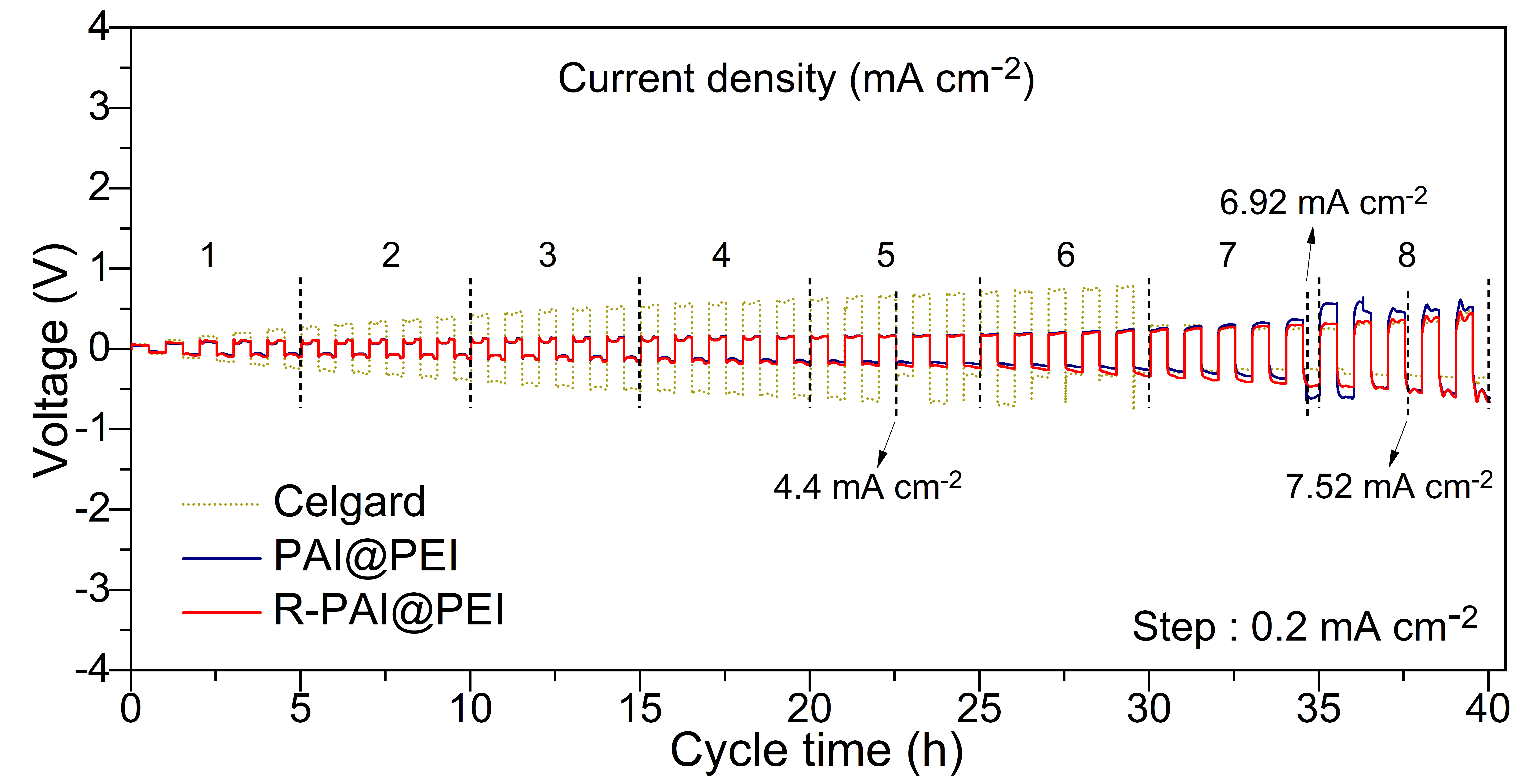


**Fig. S26** The critical current density of Li||Li symmetric cells, ranging from 0 to 8 mA cm^−2^ with increments of 0.2 mA cm^−2^


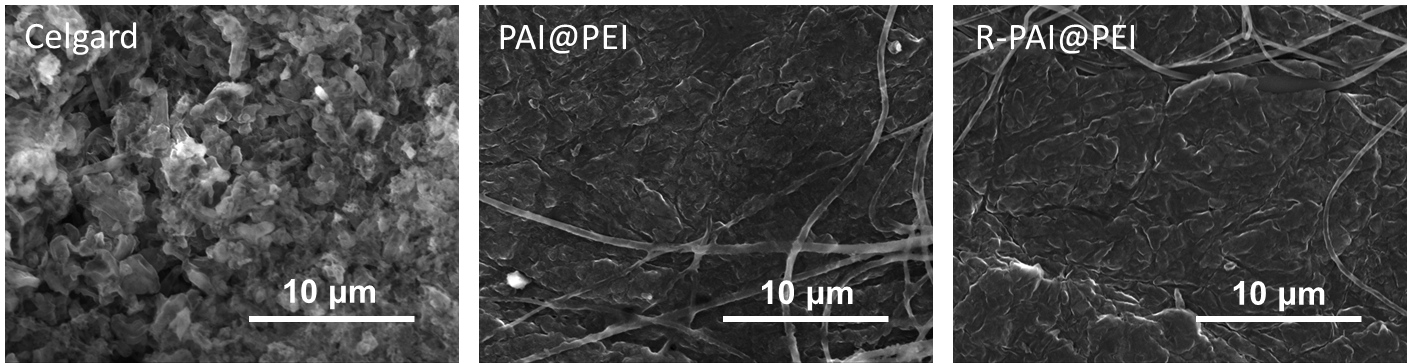


**Fig. S27** Morphology of the deposited lithium on Li anode of Li||Cu cells with the Celgard, PAI@PEI, and R-PAI@PEI separators after 10 cycles


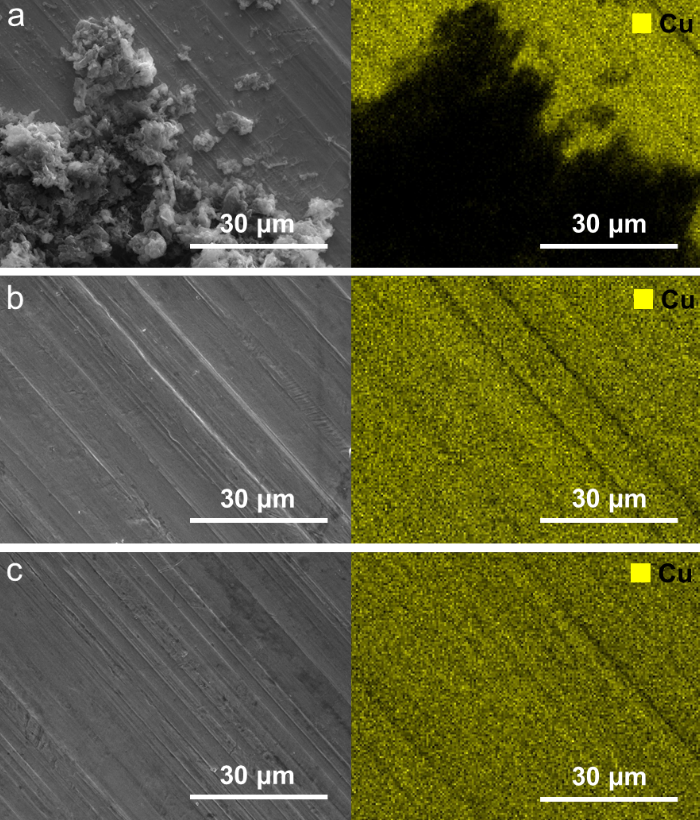


**Fig. S28** Post-cycling SEM images of the Cu foil of Li||Cu cells with the **a** Celgard, **b** PAI@PEI, and **c** R-PAI@PEI separators


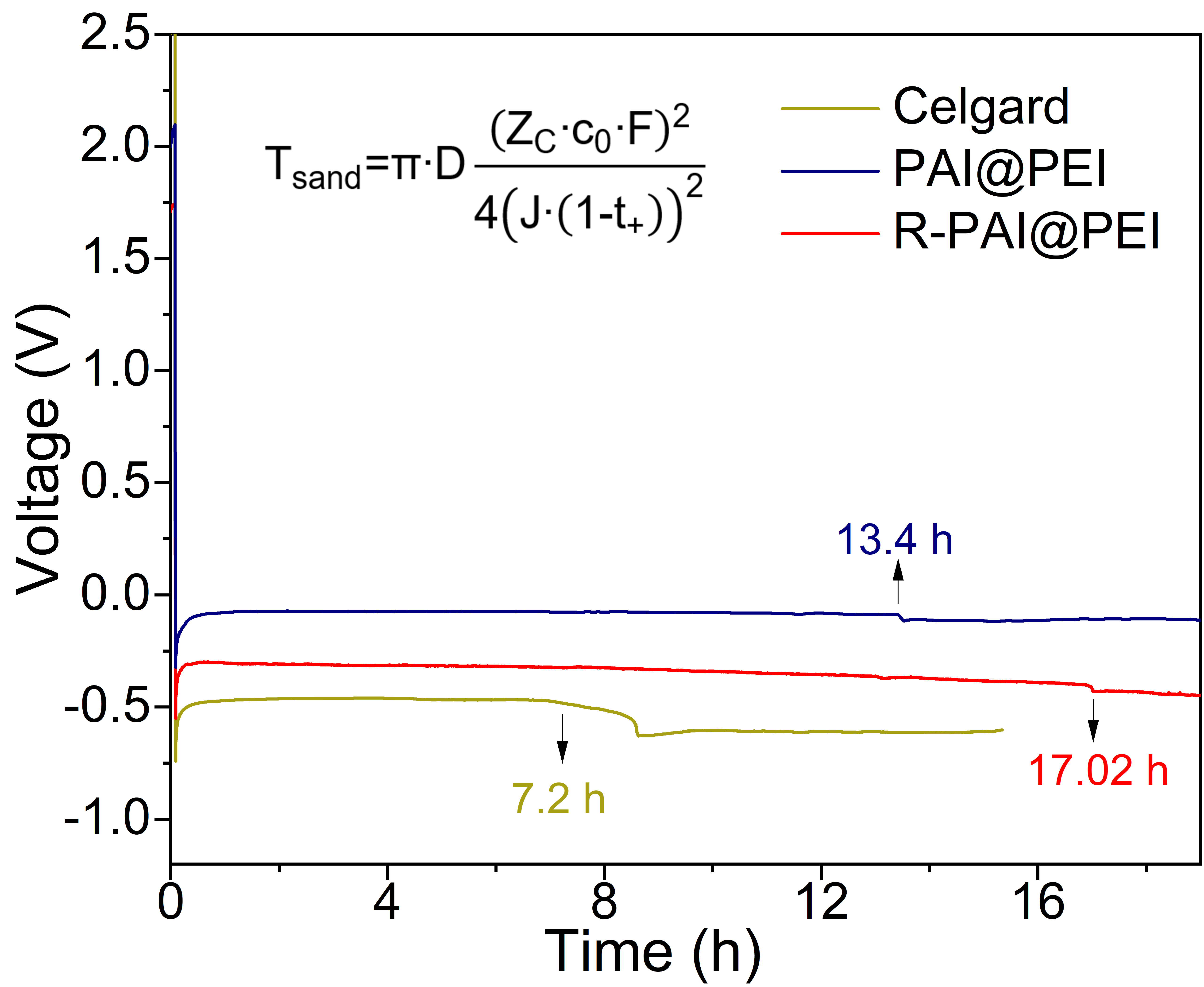


**Fig. S29** Voltage-time curves of Li||Cu cells with different separators at a current density of 4 mA cm^−2^ (D, Z_C_, F, J, c_0_, and t_+_ corresponding to the diffusion coefficient of Li^+^, charge, Faraday constant, current density, concentration of Li^+^, and transfer number, respectively.)


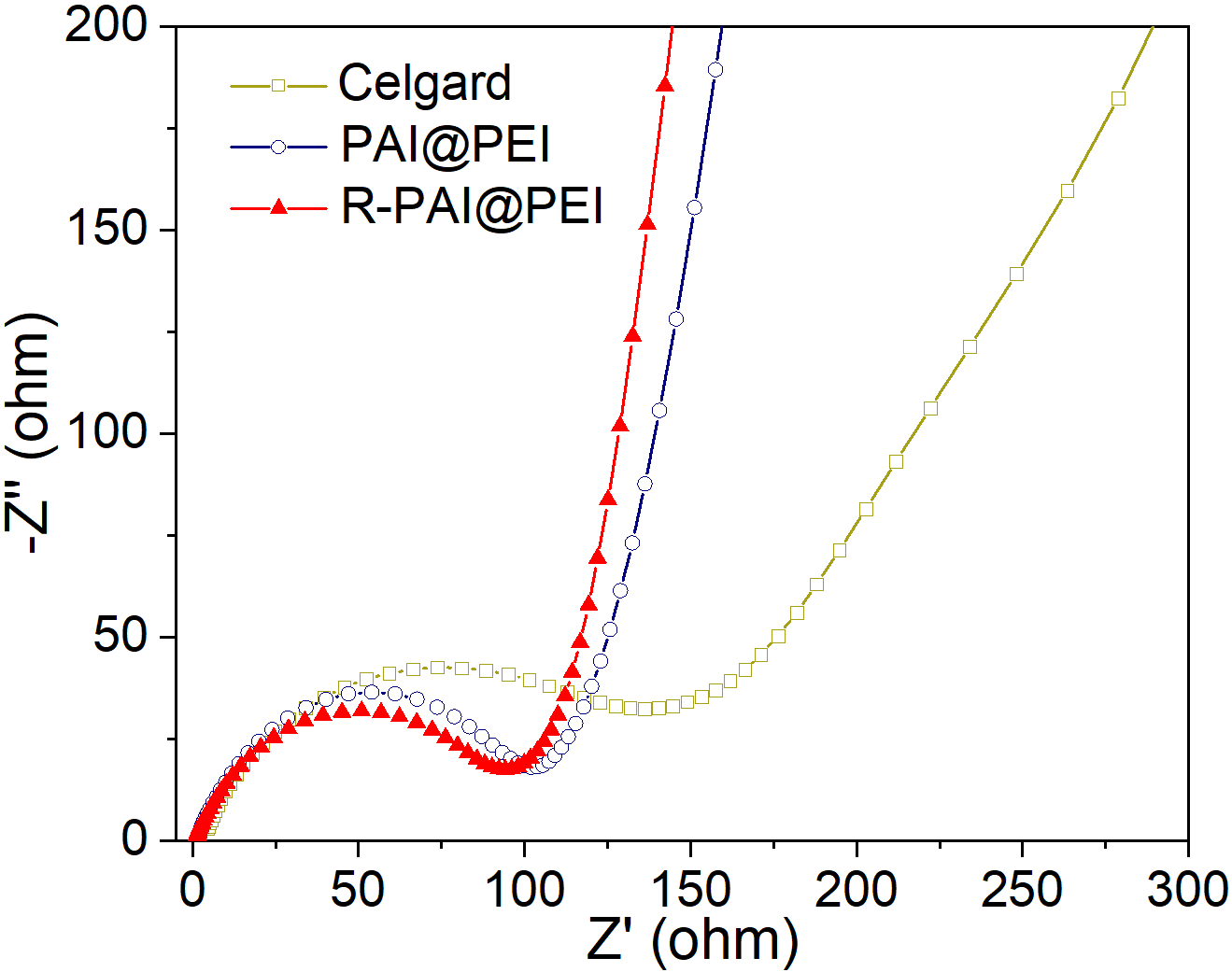


**Fig. S30** The EIS of Li||NCM523 cells with the Celgard, PAI@PEI, and R-PAI@PEI separators

**
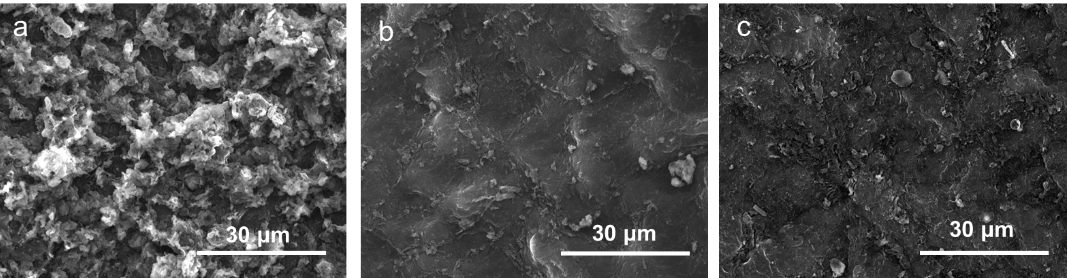
**

**Fig. S31** SEM images of Li metal in Li||NCM523 cells with the **a** Celgard, **b** PAI@PEI, and **c** R-PAI@PEI separators


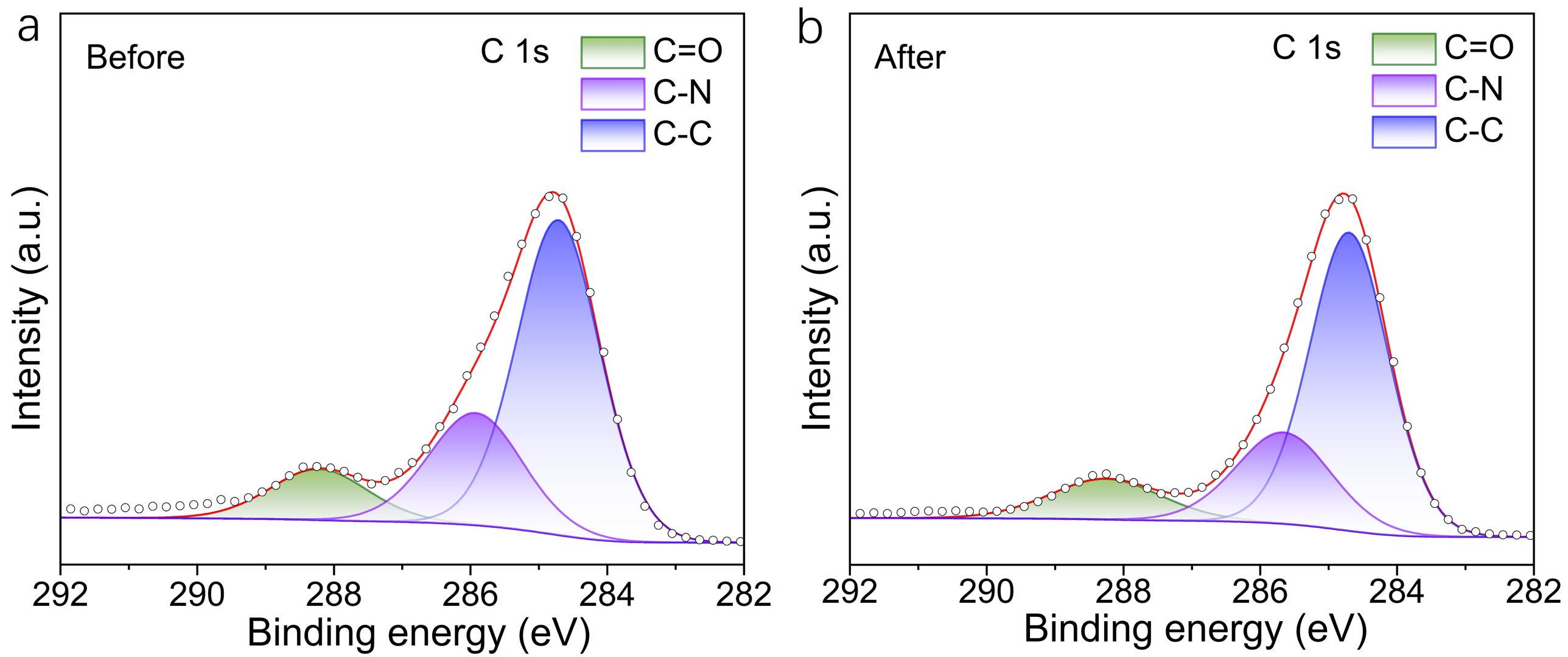


**Fig. S32** The XPS of R-PAI@PEI separator before and after cycling of Li||NCM523 cells

**Table S1** The physical properties and ionic conductivity of the sample

| **Samples** | **Puncture strength/N** | **Gurley numbers/s** | **Porosity** | **Ionic conductivity**  **/mS cm^-1^** |
| --- | --- | --- | --- | --- |
| Celgard | 5.03 | 191.0 | 40.7% | 0.79 |
| **I**PAI@PEI | 1.768 | 2.3 | 88.3% | 1.60 |
| R-PAI@PEI | 1.950 | 6.5 | 73.0% | 1.63 |
| 2R-PAI@PEI | 1.765 | 6.6 | 72.5% | 1.52 |
| 3R-PAI@PEI | 1.659 | 7.1 | 70.8% | 1.44 |

**Table S2** Comparison of separators with irreversible thermal shutdown function between PAI@PEI and reported separators

| **Year** | **Samples** | **Preparation**  **method** | **Shutdown temperature (℃)** | **The recoverability of the pores** | **References** |
| --- | --- | --- | --- | --- | --- |
| 2025 | **PAI@PEI** | **Coaxial electrospinning** | **400** | **Restorability** | **This work** |
|  | DCS | Coaxial Electrospinning with decosane-based micro capsule | 120 | Nonrecovery | [S8] |
|  | CF@HAP | In situ growth and dip-coating | 60 | Nonrecovery | [S9] |
|  | VC-(DMFu)_m_ | Uniform solution after mixing | 120 | Nonrecovery | [S10] |
|  | PCIE | In situ polymerization | 120 | Nonrecovery | [S11] |
|  | TS | Coaxial Electrospinning and coating | 105 | Nonrecovery | [S12] |
| 2024 | EVB | Coaxial Electrospinning | 110 | Nonrecovery | [S13] |
|  | PBO-BN/PVDF | Filming and coating process | 100 | Nonrecovery | [S14] |
|  | DFM | Utilizing hydrophobic association | 172 | Nonrecovery | [S15] |
|  | PES | Electrospining | 120 | Nonrecovery | [S16] |
|  | PNIPAM/PP | Grafting via the Michael addition reaction | 65 | Nonrecovery | [S17] |
| 2023 | Na-Alg/PMIA | Non-solvent phase induced separation | 200 | Nonrecovery | [S18] |
|  | CUIA-PE | In-situ radical andom polymerization | 170 | Nonrecovery | [S19] |
|  | PVDF/PEI | Solution casting | 150 | Nonrecovery | [S20] |
|  | BM/PAN | Electrospinning technique | 170 | Nonrecovery | [S21] |
|  | DEE | Mixing | 150 | Nonrecovery | [S22] |
| 2022 | EVAs/PP | Coating | 85 | Nonrecovery | [S23] |
|  | HA-1:3/PE | Liquid-phase coating | 130 | Nonrecovery | [S24] |
|  | PCM-TEP@SiO₂/PP | Microcapsules and coating | 80 | Nonrecovery | [S25] |
|  | GPE | Radical polymerization and phase separation | 300 | Nonrecovery | [S26] |
|  | PP-Al_2_O_3_-PE | Multilayer co-extrusion and sequential bidirectional drawing | 130 | Nonrecovery | [S27] |
| 2021-2017 | PSF-b-PEG | Selective swelling | 125 | Nonrecovery | [S28] |
|  | PBEI | Electrospinning and in situ welding | 235 | Nonrecovery | [S29] |
|  | PPE | Solution-mixing | 130 | Nonrecovery | [S30] |
|  | CPC | Paper-making and lamination | 200 | Nonrecovery | [S31] |
|  | TPP@PVDF-HFP | Coaxial electrospinning | 160 | Nonrecovery | [S32] |

**Table S3** Comparison of cycle life and the Li^+^ transference number of Li||Li cells between R-PAI@PEI and reported separators

| **Samples** | **Current density (mA cm^-1^)** | **Area capacity (mAh cm^-1^)** | **Cycling time (h)** | **Li-ion transfer number (*t*_Li_^+^)** | **References** |
| --- | --- | --- | --- | --- | --- |
| **R-PAI@PEI** | **1** | **1** | **760** | **0.71** | **This work** |
| PI-based PI/PIL | 0.5 | — | 250 | 0.69 | [S33] |
| PI-based PI-SO_3_ | 1 | — | 360 | 0.63 | [S34] |
| PI-based Mesoporous PI | 4 | 4 | 500 | — | [S35] |
| PI-based TEPI-PVP | 2 | 2 | 400 | 0.65 | [S36] |
| PI-based PBI@PI-COF10 | 1 | 1 | 500 | 0.53 | [S37] |
| PP-based Ti_0.87_O_2_/PP | 2 | 1 | 300 | 0.55 | [S38] |
| PP-based BFO/PP | 1 | 1 | 350 | — | [S39] |
| PP-based PZT/PP/PZT | 2 | 2 | 200 | — | [S40] |
| PP-based PPNFS-120 | 0.5 | 0.5 | 500 | 0.55 | [S41] |
| PP-based PPTA/LLZTO | 0.5 | 1 | 500 | 0.57 | [S42] |
| PP-based MOFs@PP | 1 | 0.5 | 150 | 0.68 | [S43] |
| PE-based SPMC@PE | 1 | 1 | 500 | 0.62 | [S44] |
| PE-based CS@TiO_2_@PE | 1 | 0.5 | 600 | 0.63 | [S45] |
| PVDF-based PC/PMT/P | 0.2 | 0.2 | 600 | 0.57 | [S46] |
| PBO-based PBO/NW | 0.38 | — | 700 | — | [S47] |
| PAN-based PEO/LiTFSI | 0.5 | 0.25 | 300 | — | [S48] |
| PEEK-based ZIF-PIO | 0.5 | — | 100 | 0.68 | [S49] |
| ANF-based mANFs-20 | 0.5 | — | 600 | 0.67 | [S50] |
| PMIA-based separator | 1 | 0.5 | 200 | 0.54 | [S51] |

**Supplementary References**

1. M.-J. Frisch, G.-W. Trucks, H.-B. Schlegel, G.-E. Scuseria, M.-A. Robb, et al., Gaussian 16, Revision A. 03, Gaussian, Inc., Wallingford CT (USA), 2016.
2. M.J. Abraham, T. Murtola, R. Schulz, S. Páll, J.C. Smith et al., GROMACS: High performance molecular simulations through multi-level parallelism from laptops to supercomputers. SoftwareX **1–2**, 19–25 (2015). <https://doi.org/10.1016/j.softx.2015.06.001>
3. J. Huang, A.D. MacKerell Jr, CHARMM36 all-atom additive protein force field: Validation based on comparison to NMR data. J. Comput. Chem. **34**(25), 2135–2145 (2013). <https://doi.org/10.1002/jcc.23354>
4. T. Darden, D. York, L. Pedersen, Particle mesh Ewald: an *N*⋅log(*N*) method for Ewald sums in large systems. J. Chem. Phys. **98**(12), 10089–10092 (1993). <https://doi.org/10.1063/1.464397>
5. G. Bussi, D. Donadio, M. Parrinello, Canonical sampling through velocity rescaling. J. Chem. Phys. **126**(1), 014101 (2007). <https://doi.org/10.1063/1.2408420>
6. M. Parrinello, A. Rahman, Polymorphic transitions in single crystals: a new molecular dynamics method. J. Appl. Phys. **52**(12), 7182–7190 (1981). <https://doi.org/10.1063/1.328693>
7. S.K. Reed, O.J. Lanning, P.A. Madden, Electrochemical interface between an ionic liquid and a model metallic electrode. J. Chem. Phys. **126**(8), 084704 (2007). <https://doi.org/10.1063/1.2464084>
8. M. Wang, W. Chen, X. Shen, W. Wang, K. Wang et al., “Hybrid bidirectional-gradient” phase change separator for battery all-temperature-range performance enhancement. Chem. Eng. J. **511**, 161940 (2025). <https://doi.org/10.1016/j.cej.2025.161940>
9. J.-L. Yang, X.-X. Zhao, H.-H. Liu, J.-M. Cao, H.-J. Liang et al., Nature-inspired separator with thermal sealing reinforcement toward sustainable sodium-ion batteries. ACS Nano **19**(16), 15983–15993 (2025). <https://doi.org/10.1021/acsnano.5c02393>
10. A. Ghosh, S. Tian, M. Zhang, I.L. Gómez, Q. Chen et al., Deciphering a new electrolyte formulation for intelligent modulation of thermal runaway to improve the safety of lithium-ion batteries. Adv. Funct. Mater. **35**(39), 2502761 (2025). <https://doi.org/10.1002/adfm.202502761>
11. L. Du, G. Xu, C. Sun, Y.-H. Zhang, H. Zhang et al., Smart gel polymer electrolytes enlightening high safety and long life sodium ion batteries. Nat. Commun. **16**, 2979 (2025). <https://doi.org/10.1038/s41467-025-57964-7>
12. Y. Zhang, R. He, H. Liu, H. Liu, X.-X. Zhang, Dual thermal-response separator utilizing phase change materials with polysulfides chemisorption for safe and stable lithium–sulfur batteries. J. Energy Storage **113**, 115647 (2025). <https://doi.org/10.1016/j.est.2025.115647>
13. K. Wang, W. Wang, Y. Wang, M. Wang, Dual phase change separator combining cooling and thermal shutdown functions for Li-ion battery with enhanced safety. Chem. Eng. J. **481**, 148538 (2024). <https://doi.org/10.1016/j.cej.2024.148538>
14. H. Lu, A. Du, X. Lin, Z. Zhang, S. Liu et al., Rationally coupling thermal tolerance, thermal conductance, and overheating-response in a separator for safe batteries. Energy Environ. Sci. **17**(20), 7860–7869 (2024). <https://doi.org/10.1039/D4EE02302A>
15. Z. Zhang, G. Li, X. Du, L. Huang, G. Kang et al., Rapid thermal shutdown of deep-eutectic-polymer electrolyte enabling overheating self-protection of lithium metal batteries. Adv. Sci. **11**(48), 2409628 (2024). <https://doi.org/10.1002/advs.202409628>
16. J. Richard, N. Solati, A. Singh, V. Meunier, Y. Toda et al., Functional composite separators with cation-trapping abilities. ACS Appl. Energy Mater. **7**(10), 4335–4346 (2024). <https://doi.org/10.1021/acsaem.4c00094>
17. L.-L. Jiang, Y.-Z. Deng, T. Luo, R. Xie, X.-J. Ju et al., A smart membrane with negative thermo-responsiveness in battery electrolyte solution. J. Membr. Sci. **692**, 122266 (2024). <https://doi.org/10.1016/j.memsci.2023.122266>
18. X. Hu, Y. Li, Z. Chen, Y. Sun, C. Duan et al., Facile fabrication of PMIA composite separator with bi-functional sodium-alginate coating layer for synergistically increasing performance of lithium-ion batteries. J. Colloid Interface Sci. **648**, 951–962 (2023). <https://doi.org/10.1016/j.jcis.2023.06.060>
19. T. Dong, H. Zhang, L. Huang, J. Ma, P. Mu et al., A smart polymer electrolyte coordinates the trade-off between thermal safety and energy density of lithium batteries. Energy Storage Mater. **58**, 123–131 (2023). <https://doi.org/10.1016/j.ensm.2023.03.013>
20. M. Luo, X. Zhang, S. Wang, J. Ye, Y. Zhao et al., A thermal-ball-valve structure separator for highly safe lithium-ion batteries. Small **20**(18), 2309523 (2024). <https://doi.org/10.1002/smll.202309523>
21. T. Gao, P. Tian, Q. Xu, H. Pang, J. Ye et al., Class of boehmite/polyacrylonitrile membranes with different thermal shutdown temperatures for high-performance lithium-ion batteries. ACS Appl. Mater. Interfaces **15**(1), 2112–2123 (2023). <https://doi.org/10.1021/acsami.2c18058>
22. J. Zhang, H. Wu, X. Du, H. Zhang, L. Huang et al., Smart deep eutectic electrolyte enabling thermally induced shutdown toward high-safety lithium metal batteries. Adv. Energy Mater. **13**(3), 2202529 (2023). <https://doi.org/10.1002/aenm.202202529>
23. Z. Wei, N. Zhang, T. Feng, F. Wu, T. Zhao et al., A copolymer microspheres-coated separator to enhance thermal stability of lithium-sulfur batteries. Chem. Eng. J. **430**, 132678 (2022). <https://doi.org/10.1016/j.cej.2021.132678>
24. Y. Xiao, A. Fu, Y. Zou, L. Huang, H. Wang et al., High safety lithium-ion battery enabled by a thermal-induced shutdown separator. Chem. Eng. J. **438**, 135550 (2022). <https://doi.org/10.1016/j.cej.2022.135550>
25. Z. Liu, Y. Peng, T. Meng, L. Yu, S. Wang et al., Thermal-triggered fire-extinguishing separators by phase change materials for high-safety lithium-ion batteries. Energy Storage Mater. **47**, 445–452 (2022). <https://doi.org/10.1016/j.ensm.2022.02.020>
26. M.-C. Long, G. Wu, X.-L. Wang, Y.-Z. Wang, Self-adaptable gel polymer electrolytes enable high-performance and all-round safety lithium ion batteries. Energy Storage Mater. **53**, 62–71 (2022). <https://doi.org/10.1016/j.ensm.2022.08.044>
27. L. Ding, N. Yan, S. Zhang, R. Xu, T. Wu et al., Low-cost mass manufacturing technique for the shutdown-functionalized lithium-ion battery separator based on Al_2_O_3_ coating online construction during the β-iPP cavitation process. ACS Appl. Mater. Interfaces **14**(5), 6714–6728 (2022). <https://doi.org/10.1021/acsami.1c22080>
28. H. Yang, X. Shi, S. Chu, Z. Shao, Y. Wang, Design of block-copolymer nanoporous membranes for robust and safer lithium-ion battery separators. Adv. Sci. **8**(7), 2003096 (2021). <https://doi.org/10.1002/advs.202003096>
29. G. Sun, B. Liu, H. Niu, F. Hao, N. Chen et al., *In situ* welding: Superb strength, good wettability and fire resistance tri-layer separator with shutdown function for high-safety lithium ion battery. J. Membr. Sci. **595**, 117509 (2020). <https://doi.org/10.1016/j.memsci.2019.117509>
30. Q. Zhou, S. Dong, Z. Lv, G. Xu, L. Huang et al., A temperature-responsive electrolyte endowing superior safety characteristic of lithium metal batteries. Adv. Energy Mater. **10**(6), 1903441 (2020). <https://doi.org/10.1002/aenm.201903441>
31. R. Pan, X. Xu, R. Sun, Z. Wang, J. Lindh et al., Nanocellulose modified polyethylene separators for lithium metal batteries. Small **14**(21), 1704371 (2018). <https://doi.org/10.1002/smll.201704371>
32. K. Liu, W. Liu, Y. Qiu, B. Kong, Y. Sun et al., Electrospun core-shell microfiber separator with thermal-triggered flame-retardant properties for lithium-ion batteries. Sci. Adv. **3**(1), e1601978 (2017). <https://doi.org/10.1126/sciadv.1601978>
33. Y.-H. Lin, W.-M. Huang, J.-Y. Li, M.G. Mohamed, C.-Y. Tseng et al., Nonsolvent-induced phase separation polyimide/polymeric ionic liquid separators for high-performance and safe lithium metal-based batteries. J. Membr. Sci. **728**, 124125 (2025). <https://doi.org/10.1016/j.memsci.2025.124125>
34. H. Huang, K. Chen, C. Li, Z. Zhou, W. Wang et al., Zwitterion grafted polyimide separator for improving lithium-ion transport and its application in LiCoO_2_ batteries. Chem. Eng. J. **481**, 148577 (2024). <https://doi.org/10.1016/j.cej.2024.148577>
35. D. Guo, L. Mu, F. Lin, G. Liu, Mesoporous polyimide thin films as dendrite-suppressing separators for lithium–metal batteries. ACS Nano **18**(1), 155–163 (2024). <https://doi.org/10.1021/acsnano.3c04159>
36. S.E. Park, K. Shin, J. Hyuk Yang, B.K. Park, S.Y. Kim et al., Track-etched polyimide separator decorated with polyvinylpyrrolidone for self-assembling a robust protective layer on lithium-metal anode. Chem. Eng. J. **445**, 136801 (2022). <https://doi.org/10.1016/j.cej.2022.136801>
37. S. Jalees, A. Hussain, R. Iqbal, W. Raza, A. Ahmad et al., Functional PBI membrane based on polyimide covalent organic framework for durable lithium metal battery. J. Energy Storage **101**, 113985 (2024). <https://doi.org/10.1016/j.est.2024.113985>
38. P. Xiong, F. Zhang, X. Zhang, Y. Liu, Y. Wu et al., Atomic-scale regulation of anionic and cationic migration in alkali metal batteries. Nat. Commun. **12**(1), 4184 (2021). <https://doi.org/10.1038/s41467-021-24399-9>
39. L. Xue, W. Chen, Y. Hu, T. Lei, C. Yang et al., Ferroelectric polarization accelerates lithium-ion diffusion for dendrite-free and highly-practical lithium-metal batteries. Nano Energy **79**, 105481 (2021). <https://doi.org/10.1016/j.nanoen.2020.105481>
40. Z. Hu, F. Liu, J. Gao, W. Zhou, H. Huo et al., Dendrite-free lithium plating induced by *in situ* transferring protection layer from separator. Adv. Funct. Mater. **30**(5), 1907020 (2020). https://doi.org/10.1002/adfm.201907020
41. Z. Zou, Y. Wei, Z. Hu, H. Pu, Synthesis of polypropylene nanofiber separators for lithium-ion batteries *via* nanolayer coextrusion. Chem. Eng. J. **474**, 145724 (2023). <https://doi.org/10.1016/j.cej.2023.145724>
42. Y. Mao, W. Sun, Y. Qiao, X. Liu, C. Xu et al., A high strength hybrid separator with fast ionic conductor for dendrite-free lithium metal batteries. Chem. Eng. J. **416**, 129119 (2021). <https://doi.org/10.1016/j.cej.2021.129119>
43. Z. Hao, Y. Wu, Q. Zhao, J. Tang, Q. Zhang et al., Functional separators regulating ion transport enabled by metal-organic frameworks for dendrite-free lithium metal anodes. Adv. Funct. Mater. **31**(33), 2102938 (2021). <https://doi.org/10.1002/adfm.202102938>
44. Z. Ji, Z. Zhu, A. Ye, J. Yang, W. Cai et al., Selectively permeable mesoporous separator coating by anti-gravity 2D-microfluidic for lithium metal batteries. Energy Storage Mater. **75**, 104005 (2025). <https://doi.org/10.1016/j.ensm.2025.104005>
45. L. Yang, X. Gao, J. Li, Y. Gao, M. Zhang et al., Anchoring carbon spheres on titanium dioxide modified commercial polyethylene (PE) separator to suppress lithium dendrites for lithium metal batteries. Small **20**(27), 2310915 (2024). <https://doi.org/10.1002/smll.202310915>
46. Y. Cui, D. Miao, G. Yu, M. Zhou, R. Liu et al., Novel quasi-solid-state composite electrolytes boost interfacial Li^+^ transport for long-cycling and dendrite-free lithium metal batteries. Energy Storage Mater. **56**, 258–266 (2023). <https://doi.org/10.1016/j.ensm.2023.01.016>
47. X. Hao, J. Zhu, X. Jiang, H. Wu, J. Qiao et al., Ultrastrong polyoxyzole nanofiber membranes for dendrite-proof and heat-resistant battery separators. Nano Lett. **16**(5), 2981–2987 (2016). <https://doi.org/10.1021/acs.nanolett.5b05133>
48. Y. Ma, J. Wan, Y. Yang, Y. Ye, X. Xiao et al., Scalable, ultrathin, and high-temperature-resistant solid polymer electrolytes for energy-dense lithium metal batteries. Adv. Energy Mater. **12**(15), 2103720 (2022). <https://doi.org/10.1002/aenm.202103720>
49. X. Yang, Z. An, P. Zhang, S. Kim, P.J. Yoo, Catalytic metal-organic framework-functionalized inverse-opal architectured polymeric separator for high-performance Li-S batteries. Adv. Funct. Mater. **35**(29), 2419983 (2025). <https://doi.org/10.1002/adfm.202419983>
50. F. Guo, M. Xu, S. Feng, K. Zhang, X. Zheng et al., SiO_2_-assisted co-assembly of ultrafine aramid nanofiber-based porous thermal-resistant separator for Lithium-ion batteries. Chem. Eng. J. **519**, 165583 (2025). <https://doi.org/10.1016/j.cej.2025.165583>
51. J. Ren, L. Gao, H. He, H. Li, Y. Su et al., Heat-resistant PMIA separator with highly interconnected pore structure for thermally stable and high energy lithium-ion batteries. J. Energy Chem. **104**, 716–725 (2025). <https://doi.org/10.1016/j.jechem.2025.01.027>
